# Patient characteristics and lifestyle determinants of quality of life among women with endometriosis: a systematic review

**DOI:** 10.1530/RAF-25-0094

**Published:** 2026-05-20

**Authors:** Zélia Breton, Giorgia Elisabeth Colombo, Nadjib Mokraoui, Yahya Mahamat-Saleh, Mathilde Pinault, Jacopo Conforti, Sabine Naudin, Maïa Alexaline, Marina Kvaskoff

**Affiliations:** ^1^ Université Paris-Saclay, UVSQ, Inserm, CESP, Villejuif, France; ^2^ Lyv Healthcare, Nantes, France; ^3^ Department of Obstetrics and Gynaecology, Chelsea and Westminster Hospital NHS Foundation Trust, London, UK; ^4^ Nutrition and Metabolism Branch, International Agency for Research on Cancer, Lyon, France; ^5^ Department of Clinical and Experimental Sciences, University of Brescia, Brescia, Italy

**Keywords:** endometriosis, interventions, lifestyle, mental health, quality of life, symptoms

## Abstract

**Abstract:**

Endometriosis is a chronic inflammatory disease presenting with debilitating symptoms strongly impacting patients’ quality of life (QoL). Assessing QoL is crucial for understanding the full patient experience beyond clinical symptoms. This narrative systematic review specifically aimed to identify and describe non-pharmacological and non-surgical factors associated with QoL in women with endometriosis as reported in the literature. Following PRISMA guidelines, we systematically searched PubMed, MEDLINE, Web of Science, and Embase up to January 15, 2025, for case–control, cohort, randomized controlled trials, and cross-sectional studies. This study was registered in PROSPERO (registration number: CRD42023438457) Eligible studies assessed socio-demographic characteristics, lifestyle factors, symptoms, comorbidities, or coping strategies in relation to QoL among women with endometriosis. Twenty-one studies with a low risk of bias were included. A better QoL was related to favorable socioeconomic profiles (*n* = 3), high care satisfaction (*n* = 2), partner involvement (*n* = 1), healthier lifestyle habits (*n* = 1), and the use of coping therapeutic strategies. Poor QoL was associated with poor health (*n* = 5), more severe symptoms (*n* = 5), unhealthy lifestyle habits (*n* = 2), and ineffective use of coping strategies (*n* = 2). This review highlights a broad range of non-medical determinants of QoL, underscoring that clinical measures alone fail to capture the full patient experience. By consolidating evidence across diverse study designs, it offers a comprehensive overview of modifiable factors that could inform holistic, patient-centered approaches to endometriosis care. Further prospective and interventional studies are needed to clarify causal pathways and evaluate the effectiveness of targeted strategies to improve QoL.

**Lay summary:**

Endometriosis is a chronic disease affecting around 10% of women of reproductive age, associated with pain, infertility, and significant impacts on daily life. Because clinical measures alone fail to capture the full patient experience, assessing QoL is essential to understand the real burden of the disease. This review examined non-pharmacological and non-surgical factors that may be associated with QoL, such as lifestyle habits, socioeconomic conditions, health status, and coping strategies. Across 21 studies, we found that a better QoL was linked to good healthcare access, healthy habits, and effective coping mechanisms. Conversely, severe symptoms, poor health, and unhealthy lifestyles (smoking and alcohol use) were associated with worse QoL. The findings highlight the need for a more holistic approach to endometriosis care, combining psychological support, lifestyle guidance, and improved access to quality healthcare. Future research should use standardized QoL tools and robust study designs to develop evidence-based strategies that can meaningfully enhance patient well-being.

## Introduction

### Rationale

Endometriosis is a chronic and inflammatory disease that affects approximately 10% of women worldwide (Zondervan *et al.* 2020). It is characterized by the presence of endometrial-like tissue outside the uterine cavity. This condition is associated with painful symptoms, infertility, and an increased risk of other chronic health issues, profoundly impacting patients’ lives (Nnoaham *et al.* 2011, Kvaskoff *et al.* 2021, 2015, Missmer *et al.* 2021). Adenomyosis, characterized by the presence of endometrium-like lesions within the myometrium, was once considered a subtype of endometriosis. However, it is now recognized as a distinct condition, although the two frequently coexist (Vannuccini & Petraglia 2019, Bulun *et al.* 2023).

Quality of life (QoL) is a multidimensional concept influenced by an individual’s perception within their cultural context and shaped by various factors. It encompasses domains such as social functioning, emotional state, mental health, and physical well-being. Health, as a key determinant of QoL, reflects the impact of disease on an individual’s life and is specifically referred to as health-related quality of life (HRQoL). Since health is a fundamental subset of QoL, QoL and HRQoL will be used interchangeably throughout this review (Torrance 1987, The WHOQOL Group 1995, Haraldstad *et al.* 2019).

Endometriosis presents a significant public health challenge, adversely affecting the QoL of patients in multiple life domains across the life course (Missmer *et al.* 2021). A lower QoL level has been consistently reported in patients with endometriosis as compared to the general population (Ruszała *et al.* 2022). Since endometriosis is a chronic systemic disease with no definitive cure (Vercellini *et al.* 2009, Taylor *et al.* 2021), it is crucial to prioritize enhancing the QoL of women living with this condition. QoL assessment is, therefore, fundamental to understanding the burden of endometriosis, as clinical measures alone fail to capture the full patient experience. Understanding QoL with endometriosis is among Australia’s top 10 research priorities (Armour *et al.* 2023).

A better understanding of the factors influencing QoL may benefit healthcare professionals’ practice by informing the development of tailored therapies and adequate support strategies. This can ultimately lead to better preventive measures and interventions to enhance patients’ QoL.

While this review refers to ‘women’, it is essential to recognize that endometriosis can affect all individuals assigned female at birth.

### Objectives

Previous reviews on the QoL of endometriosis patients have primarily focused on describing QoL levels (Bourdel *et al.* 2019) or on the medical or surgical factors associated with QoL (Gao *et al.* 2006, Deguara *et al.* 2012, Fritzer *et al.* 2012, Jia *et al.* 2012, Tan *et al.* 2013, Arcoverde *et al.* 2019, Barbara *et al.* 2021, Ambacher *et al.* 2022, Hernández *et al.* 2022, Momenimovahed *et al.* 2024, Maguire *et al.* 2025).

Other studies have explored non-pharmacological or non-surgical factors but were mostly limited to randomized controlled trials (RCTs) (Abril-Coello *et al.* 2023), specific factor types (e.g. psychosocial factors, physical therapy, pain management (Rossi *et al.* 2021, Kalfas *et al.* 2022, Abril-Coello *et al.* 2023)), or single QoL domains rather than the overall concept (Cano-Herrera *et al.* 2024). Some reviews focused on chronic pelvic pain (CPP) rather than endometriosis itself (Cheong *et al.* 2014) or did not follow a systematic methodology (La Rosa *et al.* 2020).

Therefore, it is essential to conduct a comprehensive examination of the factors influencing QoL, beyond surgical and pharmacological interventions, in order to deepen our understanding of other possible interventions that may reduce the impact of endometriosis on individuals’ lives.

This work aims to describe the non-pharmacological and non-surgical factors associated with QoL in women with endometriosis in the literature.

## Methods

### Registration information

This systematic review was conducted in adherence to the PRISMA (Preferred Reporting Items for Systematic Reviews and Meta-Analyses) guidelines, ensuring methodological rigor and transparency. The review protocol was registered in the PROSPERO database (registration number: CRD42023438457). Any protocol amendments are detailed in subsequent sections.

### Eligibility criteria

This review included peer-reviewed case–control, cohort, cross-sectional studies, and trials published in English investigating factors associated with QoL or HRQoL among women diagnosed with endometriosis, regardless of the diagnostic method. To be eligible, studies had to provide QoL or HRQoL scores/measures and to examine the factors potentially associated with patients’ QoL. Lifestyle, socioeconomic and demographic characteristics, disease characteristics, symptoms, health status, menstrual health, and comorbidities were considered as exposures; in contrast, medical and surgical treatments were not considered in this review. Eligible studies had to involve women diagnosed with endometriosis and include only human participants.

Exclusion criteria included non–research-based articles (e.g. case reports, commentaries, editorials) and non–peer-reviewed gray literature, as our review targeted peer-reviewed empirical studies only. Reviews (reference lists screened), studies involving women with adenomyosis only, those with a healthy control group, and studies assessing QoL through incomplete or specific QoL scores (e.g. sexual QoL, gastrointestinal QoL, UFS QoL) were also excluded. Validation studies for QoL measures and investigations focusing solely on medical treatments or surgical interventions as factors affecting QoL were also excluded. Studies providing only abstracts or limited information were not included.

### Information sources

The PubMed, MEDLINE, Web of Science, and Embase databases were systematically searched to identify eligible studies, covering all available records from inception until January 15, 2025.

### Search strategy

The search terms included (“endometriosis” OR “adenomyosis” OR “pelvic pain” OR “dysmenorrhea”) AND (“quality of life” OR “QoL” OR “HRQoL” OR “SF-36” OR “SF-12” OR “NHP” OR “WHOQOL-BREF” OR “DUKE” OR “EQ-5D” OR “15D” OR “EHP-30” OR “EHP-5”) – see the list of abbreviations for details. One of the authors (ZB) also searched for references to similar systematic and narrative reviews in addition to searching by keyword in the databases.

### Selection process

Two authors (ZB and GC) independently conducted the study selection process. Initial screening was performed based on titles and abstracts, followed by a review of the full-texts to determine eligibility. The Rayyan software was used to streamline the screening process, employing keywords to efficiently identify studies that met the inclusion and exclusion criteria (Ouzzani *et al.* 2016). The authors worked independently and then discussed any cases of disagreement with a third investigator (MK).

### Data collection process

Data extraction was conducted independently by two team members for each article (ZB and NM, GC and JC, MA and MP). Extracted data from the final selected studies were systematically stored following standardized extraction practices. The extracted information included:

- Article details: title, first author’s last name, and year of publication.
- Study design: objectives, research questions, study period, study design, country where the study was conducted, and sampling source.
- Inclusion criteria: method of endometriosis diagnosis, stage, type, body site of endometriosis, and type of treatment.
- Outcomes: QoL scale used and the domains assessed within the scale.
- Methods: ability to evaluate the temporality of associations, variables adjusted for in the analysis, and information used for risk-of-bias assessment.
- Factors investigated: non-pharmacological and non-surgical factors potentially associated with QoL.
- Results: sample size, number of cases exposed to interventions, response rate, age range, key findings, odds ratios (ORs), relative risk (RR), or β (beta) coefficients for associated factors, and 95% confidence intervals (CIs) or *P*-values, or correlation coefficient.

Requests for additional information were sent to the respective authors for studies with missing data. Any disagreement in the extraction between the reviewers was discussed within the team. All extracted data were consolidated into a comprehensive table created with Microsoft Excel.

### Data items

Data from the articles were extracted according to the study population and/or control group.

### Study risk-of-bias assessment

The risk-of-bias assessment was conducted concurrently with data extraction by the same pairs of authors working independently (ZB and NM, GC and JC, MA, and MP). Various standardized tools were used based on the study design:

- ROBINS-E for observational non-randomized studies, assessing seven domains with results classified as low, some concerns, high, or very high risk of bias (Higgins *et al.* 2024).
- ROBINS-I for non-randomized interventional studies, evaluating seven domains with outcomes categorized as low, moderate, serious, or critical risk of bias (Sterne *et al.* 2016).
- ROB-2 for RCTs, focusing on five domains with outcomes designated as low, some concerns, or high risk of bias (Sterne *et al.* 2019).

The methodological quality of the included studies was assessed and documented using Microsoft Excel. Of note, based on this assessment, we elected to base our narrative synthesis of findings on studies with a low risk of bias or with some concerns, in order to report results from high-quality studies only. The figures detailing risk-of-bias scores were constructed using the Robvis tool (McGuinness & Higgins 2021).

### Effect measures

For each study, we reported the effect measures provided in the result section of the article. Effect estimates included ORs, RRs, or regression coefficients with their respective 95% CIs and *P*-values, mean or median differences with standard deviations (SD) and *P*-values, or correlation coefficients with *P*-values.

### Synthesis methods

The primary outcome of this review was the QoL among women with endometriosis. A narrative synthesis was conducted to summarize existing findings on factors associated with QoL. This synthesis included a detailed review of the articles, their study designs, populations, reported QoL levels, and the factors associated with QoL. Results were grouped based on the type of factors explored, including socio-demographic characteristics, healthcare pathway, health status, mental health, endometriosis symptoms, partner involvement, lifestyle, coping strategies, and non-pharmacological and non-surgical interventions.

Each group included fewer than three studies with comparable instruments, methodologies, and statistical approaches used (see Supplementary Table 1 (see section on Supplementary materials given at the end of the article)), therefore limiting the ability to use a meta-analytical approach for each group. A narrative synthesis was thus conducted; when multiple studies investigated the same factor, their results were narratively compared in order to identify consistencies and discrepancies.

## Results

### Study selection

**Figure 1:**
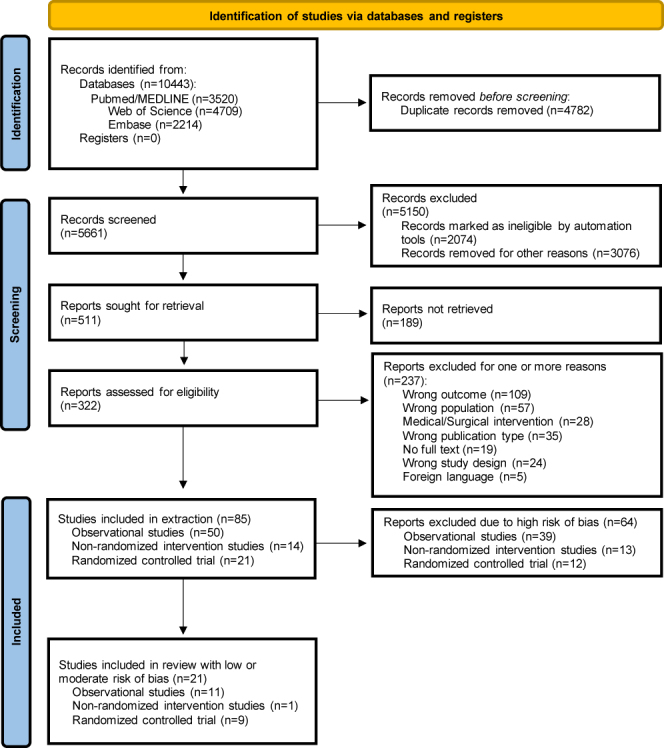
Flow diagram.

**Figure 2:**
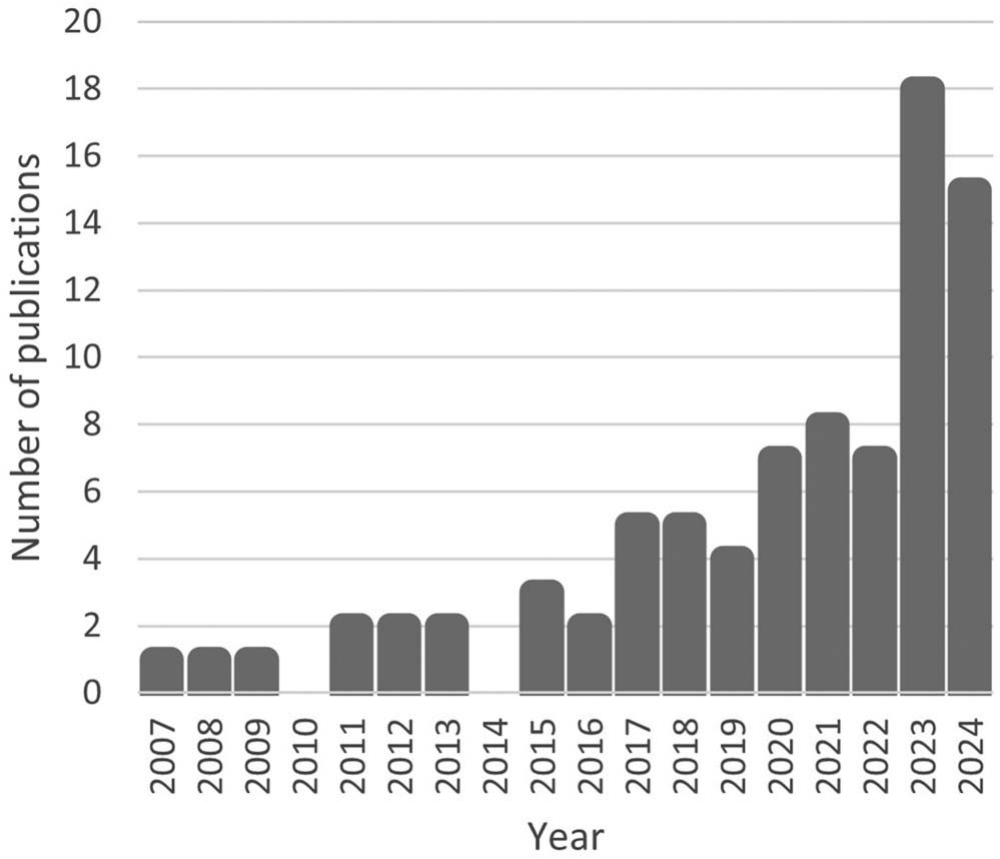
Distribution of the articles included for data extraction according to their year of publication.

The search and selection process is detailed in the flow diagram presented in Fig. 1. Database searches identified 10,443 studies. After removing 4,782 duplicates, 5,661 records were screened. A total of 511 reports were sought for retrieval, and 322 were assessed for eligibility. Applying inclusion and exclusion criteria, 237 reports were excluded, leaving a final list of 85 studies included in the extraction process. The distribution of the 85 articles included for data extraction according to their year of publication is shown in Fig. 2.

### Risk-of-bias assessment

The risk of bias for observational studies was assessed using the ROBINS-E tool, with results shown in Supplementary Fig. 1. A total of 25% of observational studies were at very high risk of bias, primarily due to exposure measurement biases (e.g. self-reported diagnoses). Over 50% were at high risk due to confounding (e.g. lack of statistical adjustments). Overall, 39 studies had high or very high risk of bias, 8 had some concerns (Apers *et al.* 2018, Mundo-López *et al.* 2020, He *et al.* 2022, Cofini *et al.* 2023, Kanti *et al.* 2024*a*,*b*, Bień *et al.* 2024, Comptour *et al.* 2024), and 3 had low risk (McPeak *et al.* 2018, O’Hara *et al.* 2021, Muharam *et al.* 2022).

The ROBINS-I tool was used for non-randomized interventional studies, with results shown in Supplementary Fig. 2. All studies except one (Breton *et al.* 2025) were rated as serious (80%) or critical (20%) risk of bias due to confounding (e.g. no statistical adjustment) and intervention classification issues (e.g. absence of control groups). None were rated as low risk.

RCTs were evaluated with the ROB-2 tool, with results presented in Supplementary Fig. 3. Up to 60% of RCTs were rated at high risk of bias, mainly due to incomplete outcome data (i.e. dropouts or attrition), although all studies reported the outcome and were thus eligible for inclusion, and due to deviations from intended interventions (e.g. lack of blinding). Eight RCTs were rated at low risk of bias (Wayne *et al.* 2008, Nodler *et al.* 2020, Artacho-Cordón *et al.* 2023, Hansen *et al.* 2023, Li *et al.* 2023, Muñoz-Gómez *et al.* 2023, Gudarzi *et al.* 2024, Rodríguez-Ruiz *et al.* 2024), and one with some concerns (Donatti *et al.* 2024).

Overall, 64 out of 85 extracted studies were classified as high risk of bias and were excluded from the narrative synthesis to base conclusions on the highest-quality studies only. The characteristics of these excluded studies are described in Supplementary Table 2.

### Characteristics of included studies

The characteristics of the 21 included studies with low or some concern risk of bias are summarized in Supplementary Table 3.

Regarding study design, 11 were observational studies (all cross-sectional), 1 was a non-randomized interventional study, and 9 were RCTs.

The studies originated from 13 countries (10 in Europe, 5 in North America, 4 in Asia, 1 in Australia, and 1 in Brazil), and were published between 2008 and 2025. Sample sizes ranged from 14 participants in an American RCT from 2008 (Wayne *et al.* 2008) to 964 participants in a 2024 French cross-sectional study (Comptour *et al.* 2024). The mean age of participants varied between 17 and 37 years.

HRQoL measurements used in the included and extracted studies are listed in Table 1. Within included studies, a total of 15 studies employed endometriosis-specific QoL scores (EHP-30: *n* = 14; EHP-5: *n* = 1), while 8 used generic QoL scores (SF-36: *n* = 5; SF-12: *n* = 2; EQ-5D: *n* = 1).

**Table 1:** Description of quality of life scores.

Type/QoL scale/domains studied	Items	Range	Interpretation
Endometriosis specific			
EHP-5		0–100	Higher scores indicate worse health status
Pain; control/powerlessness; emotional well-being; social support; self-image	Core part: difficulty in walking; lack of control or being powerless; mood swings; lack of social support; self-image		
	Modular part: difficulties with work: difficulties with intercourse; worries about infertility; worries about treatment; relationships with children; relationships with medical professionals		
EHP-30		0–100	Higher scores indicate worse health status
Pain; control/powerlessness; emotional well-being; social support; self-image	Core part: pain; control and powerlessness; emotional well-being; social support; self-image		
	Modular part: work; relationship with children; sexual intercourse; infertility; medical profession; treatment		
Endometriosis not specific			
SF-12		0–100	Higher scores indicate a better health perception
PCS; MCS	General health; physiological function; role-physical; bodily pain; mental health; vitality; social function; role-emotional		
SF-36		0–100	Higher scores indicate a better QoL
PCS; MCS	Physical functioning; role limitations due to physical health; role limitations due to emotional problems; pain; general health; vitality; social functioning; emotional well-being		
WHOQOL-BREF		0–100	Higher scores denote a higher QoL
Psychological; physical; social relationship; environment	26 questions		
WHO-QOL-100			
Psychological; physical; social relationship; environment			
EuroQol VAS		VAS: 1–100	Higher scores indicate better health status
EQ-5D	Mobility; self-care; daily activities; pain; emotional well-being	0–100	Higher scores indicate worse health status

QoL, quality of life; VAS, visual analog scale; PCS, physical component summary; MCS, mental component summary.

Regarding statistical methodology, 11 studies used regression models to analyze factors associated with QoL (e.g. ORs, β, CIs). Ten studies reported mean or median differences with SD, and 1 used correlation analyses.

The studies explored between 1 and 10 factors potentially associated with QoL, which were categorized as follows:

- Socio-demographic characteristics (*n* = 4 studies): included age, educational level, relationship status, perception of financial situation or income, access to health insurance, and occupational status, studied only in observational studies using specific and non-specific QoL scores.
- Healthcare pathway (*n* = 3 studies): factors included care satisfaction and consultations with complementary practitioners, studied in observational studies.
- Health status (*n* = 5 studies): factors included pregnancy, BMI, comorbidities, stage and type of endometriosis, and sleep quality, assessed in observational studies using non-specific QoL scores.
- Mental health (*n* = 3 studies): factors such as anxiety and depression level, pain catastrophizing, psychological morbidity, and anger were evaluated in observational studies.
- Endometriosis symptoms (*n* = 5 studies): included painful symptoms, fatigue, and duration of symptoms studied in observational studies using specific and non-specific QoL scores.
- Partner involvement (*n* = 1 study): relationship impact assessed with non-specific QoL scores in an observational study.
- Lifestyle (1 study): factors included smoking, physical activity, and alcohol consumption studied in one observational study, using a non-specific QoL score.
- Coping strategies (*n* = 3 studies): included self-care strategies, self-efficacy, social support, and coping mechanisms, evaluated in observational studies with specific and non-specific QoL scores.
- Non-pharmacological and non-surgical interventions (*n* = 10 studies): factors such as dietary supplements (omega 3, vitamin D, and curcumin), innovative devices, acupuncture, and psychological, physical, and digital therapy were assessed in interventional studies (randomized or not) using specific and non-specific QoL scores. All these non-pharmacological and non-surgical interventions were assessed versus placebo or control groups at follow-up.

### Results of individual studies

The level of QoL and associated factors found in the included studies are detailed in Supplementary Table 3.

### QoL levels

Baseline QoL levels varied considerably across studies.

For endometriosis-specific QoL (EHP scale), where 100 represents the worst QoL, scores ranged from 29.3 in a study from the Netherlands (Apers *et al.* 2018) to 63 in a Spanish study (Rodríguez-Ruiz *et al.* 2024). For SF-36/SF-12, where 100 represents the best QoL, physical component scores ranged from 39 in an Italian study (Cofini *et al.* 2023) to 47 in an American study (Nodler *et al.* 2020), while mental component scores ranged from 35 to 45, respectively, in the same studies.

### Factors associated with QoL

**Figure 3:**
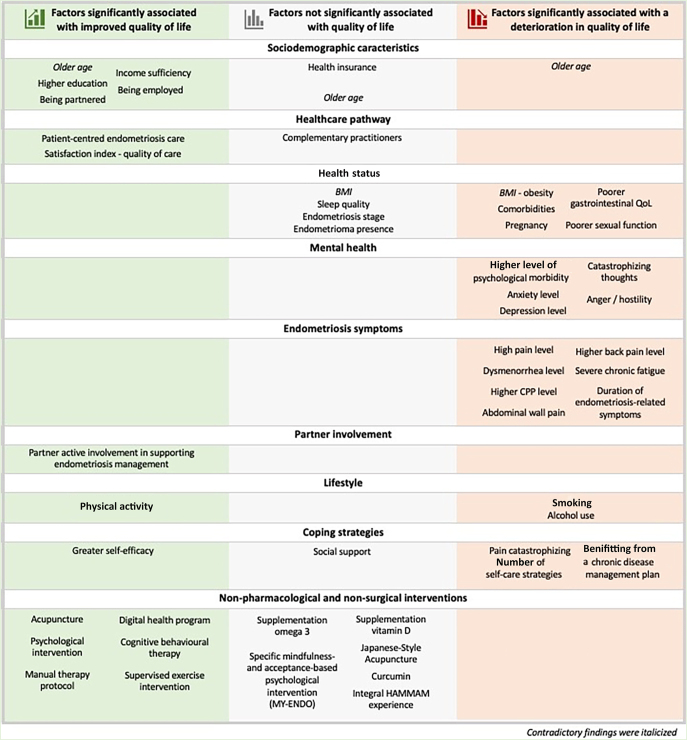
Determinants of endometriosis quality of life.

Figure 3 summarizes the factors positively, inversely, or not associated with QoL among women with endometriosis among the included studies.

#### Socio-demographic characteristics

Several socio-demographic factors were found to be associated with QoL in women with endometriosis in cross-sectional studies.

Higher education, being in a relationship, higher income, and being employed were associated with a better QoL (McPeak *et al.* 2018, O’Hara *et al.* 2021, Cofini *et al.* 2023).

The four studies investigating the association between age and QoL yielded contradictory findings: 2 reported a positive association, 1 reported no association, and 1 reported both positive and inverse associations.

McPeak *et al.* used the EHP-30, a disease-specific QoL score, and found a significantly positive association between older age and better QoL (*n* = 236, *β* = −0.13, *P* = 0.039). Similarly, two studies using the SF-36, a general QoL score, identified a positive association between older age and better mental QoL (*β* = 0.185, *P* < 0.001 and *β* = 0.2, *P* = 0.012, respectively), despite differences in overall QoL between cohorts. O’Hara *et al.* reported that the QoL of 620 Australian patients was higher than that of the 875 Italian women in Cofini *et al.*’s study. Conversely, deterioration in the physical component of QoL, measured through non-specific scores, was associated with older age in the study by O’Hara *et al.* (2021). Also, age was not associated with non-specific QoL scores in Comptour *et al.* (2024).

Among the two studies investigating the association between education and QoL, both reported a positive association. O’Hara *et al.* and Cofini *et al.*, using the SF-36, indeed observed that higher education was associated with improved QoL, but in different domains (O’Hara *et al.* 2021, Cofini *et al.* 2023). O’Hara *et al.* observed a positive association between university education and the mental component of QoL (*β* = 1.832, *P* < 0.05), while Cofini *et al.* found that a high educational level was positively associated with the physical component of QoL (*β* = 3.2, *P* < 0.001).

Finally, having a health insurance was not associated with non-specific QoL scores (O’Hara *et al.* 2021).

#### Healthcare pathway

For healthcare pathways, cross-sectional studies found positive associations between QoL and patient-centered endometriosis care, as well as satisfaction with the quality of care, using both specific and non-specific QoL scores (Apers *et al.* 2018, Cofini *et al.* 2023); whereas complementary practitioner consultations were not associated with QoL (O’Hara *et al.* 2021).

#### Health status

Health factors, such as obesity, comorbidities (yes vs no), pregnancy, poorer gastrointestinal QoL, and reduced sexual function, were associated with a lower QoL (Mundo-López *et al.* 2020, Cofini *et al.* 2023). In contrast, sleep quality, endometriosis stage, and the presence of endometrioma were not associated with QoL (Mundo-López A *et al.* 2020, Kanti *et al.* 2024*a*, Comptour *et al.* 2024).

Of the two studies that explored the association between BMI and QoL, one using the EHP-30 scale found an inverse association (Muharam *et al.* 2022), while the other using the SF-36 scale found no association (Cofini *et al.* 2023).

#### Mental health

Mental health challenges, including psychological morbidity, anxiety, depression, catastrophizing thoughts, and anger/hostility, were associated with lower QoL (Mundo-López *et al.* 2020, He *et al.* 2022, Muharam *et al.* 2022). The studies reporting this association include a Chinese study that used the SF-12 (He *et al.* 2022), as well as a Spanish study and an Indonesian study that used the EHP-30 (Mundo-López *et al.* 2020, Muharam *et al.* 2022).

#### Endometriosis symptoms

Endometriosis symptoms, particularly high pain levels, dysmenorrhea, CPP, abdominal wall pain, back pain, severe chronic fatigue, and duration of symptoms, were associated with poorer QoL (McPeak *et al.* 2018, Mundo-López *et al.* 2020, O’Hara *et al.* 2021, Muharam *et al.* 2022, Kanti *et al.* 2024*b*).

The association between higher pain levels and poorer QoL was highlighted in the Australian study by O’Hara *et al.*, which used the SF-36 (O’Hara *et al.* 2021), and in three studies using the EHP-30: an Indonesian study (*n* = 160) (Muharam *et al.* 2022), a Spanish study (*n* = 230) (Mundo-López *et al.* 2020), and a Canadian study (*n* = 352) (Kanti *et al.* 2024*b*). Since lower scores indicate better QoL, Kanti *et al.* observed the highest QoL levels, followed by Muharam *et al.*, while Mundo-López *et al.* reported the poorest QoL among participants. Muharam *et al.* found a significant association with an OR of 13.33 (*P* < 0.001, for pain evaluated as a visual analog scale and categorized as high (≥7) or low (<7)), while Mundo-López *et al.* reported an association using *β* coefficients (*β* = 1.39, *P* = 0.001, for pain evaluated with three categories). Finally, Kanti *et al.* (2024*b*) determined that the difference in QoL between women with more severe and frequent symptoms versus those with milder and less frequent symptoms was significant (*P* < 0.001).

#### Partner involvement

O’Hara *et al.* found that an active involvement of the partner in supporting endometriosis management was associated with a better QoL measured with a non-specific score (O’Hara *et al.* 2021).

#### Lifestyle

Lifestyle factors associated with a better QoL included physical activity (Cofini *et al.* 2023), observed primarily with non-specific QoL scores. In contrast, smoking and alcohol use were associated with lower QoL (Cofini *et al.* 2023).

#### Coping strategies

Concerning coping strategies, the Australian study showed that greater self-efficacy, i.e. a stronger belief in one’s ability to manage one’s health, was positively associated with non-specific QoL scores (O’Hara *et al.* 2021). In contrast, pain catastrophizing, a higher number of self-care strategies, and chronic disease management plans were associated with reduced QoL (McPeak *et al.* 2018, O’Hara *et al.* 2021). Of note, in Australia, chronic disease management is supported by the introduction of a chronic disease management plan, drawn up by the GP to structure care, set therapeutic objectives, and coordinate the involvement of different healthcare professionals. This plan enables regular medical follow-up and access to paramedical care partially reimbursed by Medicare, as part of a multidisciplinary approach. Finally, coping strategies such as social support (Mundo-López *et al.* 2020) were not associated with QoL.

#### Non-pharmacological and non-surgical interventions

Of the studies investigating the association between non-pharmacological and non-surgical interventions and QoL (*n* = 10), 6 reported a positive association, and 5 reported no association. Studies reported improvements associated with acupuncture (Li *et al.* 2023), psychological interventions (Hansen *et al.* 2023), manual therapy protocols (Muñoz-Gómez *et al.* 2023), a supervised exercise intervention (Artacho-Cordón *et al.* 2023), a digital health program (Breton *et al.* 2025), and cognitive behavioral therapy (Donatti *et al.* 2024). However, many interventions, including omega-3 or vitamin D supplementation (Nodler *et al.* 2020), mindfulness-based interventions (MY-ENDO) (Hansen *et al.* 2023), Japanese-style acupuncture (Wayne *et al.* 2008), curcumin (Gudarzi *et al.* 2024), and integral HAMMAM experience (Rodríguez-Ruiz *et al.* 2024), showed no association with QoL.

## Discussion

This systematic literature review aimed to identify non-pharmacological and non-surgical factors associated with QoL in women with endometriosis. Twenty-one studies with low risk of bias or some concerns only were included and showed that higher socio-demographic level, greater satisfaction with care, lower impact of the disease, healthy lifestyle habits, effective coping strategies, and specific non-pharmacological and non-surgical interventions were linked to better QoL. In contrast, poor health status, mental health challenges, severe endometriosis symptoms, unhealthy lifestyles, and maladaptive coping strategies were associated with lower QoL levels.

### Comparison with prior work

#### Socio-demographic characteristics

Socio-demographic factors play an important role in shaping QoL in women with endometriosis. Our findings are consistent with evidence indicating that financial strain can significantly reduce QoL across multiple domains (Bień *et al.* 2025). Employment status is also a key determinant: beyond its effect on pain or symptom management, professional engagement contributes to social integration and supports overall well-being, independently influencing HR-QoL (Vibert *et al.* 2025). However, evidence remains limited for some variables such as relationship status or household income, highlighting the need for further research in these areas. Overall, these findings underscore that socio-demographic factors should be considered alongside clinical and psychological determinants when assessing QoL and planning comprehensive care for women with endometriosis. A higher financial and social level was globally associated with better QoL in the included studies (O’Hara *et al.* 2021). Healthcare inequalities remain a major issue in chronic illnesses, emphasizing the need for equitable access to quality care pathways. Findings on age and QoL were inconsistent, with some results linking older age to better mental health (McPeak *et al.* 2018) while others associated it with worse physical well-being (O’Hara *et al.* 2021).

#### Healthcare pathway

Care satisfaction was a key determinant of QoL in our review. Meeting patients’ expectations – particularly regarding the information and support provided – positively influences their physical, psychological, and social well-being (Szlenk-Czyczerska *et al.* 2022). Adopting patient-centred care approaches, as highlighted by patient satisfaction assessments such as the Patient Assessment of Chronic Illness Care (PACIC), further supports this conclusion (Akan & Kartal 2023).

#### Health status

Poor health status, including comorbidities, lower gastrointestinal QoL, and poorer sexual function, was strongly linked to lower QoL. This is consistent with findings in other chronic diseases such as obesity, hypertension, and depression (Galenkamp *et al.* 2019). However, the association between QoL and BMI in women with endometriosis was not always consistent.

The contradictory findings regading age and BMI may be explained by several methodological elements. Depending on the study, age was associated with better, worse or no significant association with QoL (McPeak *et al.* 2018, O’Hara *et al.* 2021, Cofini *et al.* 2023, Comptour *et al.* 2024), while BMI was associated with worse QoL or no association with QoL (Muharam *et al.* 2022, Cofini *et al.* 2023). These discrepancies may arise from the use of different measurement tools (specific questionnaires such as the EHP-30 or generic ones such as the SF-36), which do not assess the same dimensions of QoL. In addition, some studies analyze physical and mental health components separately, which may be differently influenced by these factors.

#### Mental health

A poor mental health was associated with a lower QoL, as is well known in the literature outside of endometriosis (Olatunji *et al.* 2007). A recent scoping review also suggested that depressive and anxiety symptoms negatively impact HRQoL in individuals with endometriosis (Colombo *et al.* 2024). The strong interplay between pain, mental health, and QoL highlights the need for psychological support alongside symptom management (Farenga *et al.* 2024). Systematic reviews consistently identified depression, anxiety, stress, and pain catastrophizing as key psychosocial factors contributing to poorer QoL (Kalfas *et al.* 2022, Maulenkul *et al.* 2024). In addition, long diagnostic delays, limited treatment efficacy, and persistent symptoms exacerbate emotional distress, underscoring the need for further research into care pathways, chronic symptom management, and social determinants such as support from surroundings and perceived equity. A meta-analysis by Wang *et al.* further reinforces the link between endometriosis, mental health disturbances, and a lower level in both mental and physical HRQoL (Wang *et al.* 2021), emphasizing the necessity of integrating psychological support into endometriosis care.

#### Endometriosis symptoms

Findings from the included studies suggest that a low QoL is strongly associated with endometriosis symptoms, particularly chronic pain, rather than with infertility. While specific to endometriosis, this pattern aligns with other chronic pain conditions, highlighting the need for effective pain management, including care delivered by pain specialists (Zis *et al.* 2019). Research consistently demonstrated the impact of pelvic pain on QoL, with a clear link between pain severity and reduced well-being (Rossi *et al.* 2021, As-Sanie *et al.* 2024). Marinho *et al.* further emphasized that QoL impact is primarily driven by symptoms rather than the disease itself (Marinho *et al.* 2018). Endometriosis also significantly affects sexual health and relationships. Patients frequently report dyspareunia, sexual dysfunction, dissatisfaction, and distress, which profoundly impact their well-being. Moreover, this can strain relationship quality and affect partners, a dual burden highlighted in Rossi’s review, stressing the need for further research into sexual dysfunction and its relational impacts to better address these challenges (Rossi *et al.* 2021). However, studies linking dyspareunia to QoL often present a high risk of bias, thus limiting their reliability.

#### Partner involvement

Higher partner involvement in disease management was associated with better QoL, highlighting the importance of support systems in coping with endometriosis. The broader impact of endometriosis on social relationships, work productivity, and economic stability is well-documented (Della Corte *et al.* 2020). While these factors clearly influence QoL among patients, more studies are needed to explore these domains comprehensively, particularly as they were not fully captured in the scope of our systematic review. Understanding these interconnected influences will be essential for developing holistic interventions that address both individual and relational dimensions of endometriosis.

#### Lifestyle

A healthy lifestyle was closely linked to a higher QoL, with regular physical activity and avoiding alcohol and tobacco being particularly beneficial. Systematic reviews such as Mazur-Bialy *et al.'s* confirm that physical activity is associated with better QoL, emphasizing its importance in managing chronic conditions (Mazur-Bialy *et al.* 2024). While this association is well-documented in the general population (Amiri *et al.* 2024) and for other chronic conditions, such as chronic obstructive pulmonary disease and multiple sclerosis (Leong *et al.* 2018, Bouloukaki *et al.* 2024), there remains a lack of good-quality research on the specific effects of nutrition, sleep, and other lifestyle factors in women with endometriosis. However, the existing evidence suggests the importance of incorporating behavior modification and lifestyle interventions into endometriosis care. Programs that promote healthier habits, including physical activity, sleep hygiene, and dietary guidance, could be pivotal in improving patients’ overall well-being and disease management. Further targeted research is needed to better understand these impacts and develop tailored prevention and intervention strategies.

#### Coping strategies

Self-efficacy and effective coping mechanisms were associated with improved QoL among women with endometriosis. In contrast, pain catastrophizing was linked to poorer QoL, emphasizing the need for psychological interventions that promote resilience. The observed association between multiple self-care strategies and lower QoL should be interpreted cautiously, as it may reflect the severity of symptoms rather than the strategies themselves being detrimental. The study of O’Hara *et al.* 2021 assessed the total number of self-care activities, without distinguishing between specific strategies, although their effectiveness varies considerably. As shown in a recent Australian study, some self-care practices (e.g. cannabis use, heat therapy, dietary changes) were perceived as helpful for reducing endometriosis-related pain, whereas others such as yoga, stretching, or even exercise were reported as less effective or sometimes associated with worsening symptoms, including increased pelvic pain or fatigue. This suggests that not all self-care strategies are beneficial, and that individuals who experiment with a greater number of strategies may do so because they experience more severe or persistent symptoms, which could in turn explain their lower QoL. Thus, the association may reflect both heterogeneous effectiveness across self-care approaches and a greater symptom burden, leading patients to adopt multiple strategies. Further research is needed to better understand the barriers and facilitators to effective self-management in women with endometriosis (O’Hara *et al.* 2019).

#### Non-pharmacological and non-surgical interventions

Non-pharmacological and non-surgical interventions such as acupuncture, psychological interventions, manual therapies, digital health programs, cognitive behavioral therapy, and supervised exercise interventions have been associated with higher levels of QoL among women with endometriosis. Physical therapy, in particular, has demonstrated benefits in reducing pain intensity and improving the physical component of QoL (Abril-Coello *et al.* 2023). While acupuncture has shown promise in enhancing QoL (Afreen *et al.* 2024), its long-term effects remain unclear, and findings on its effectiveness are mixed. A meta-analysis (Giese *et al.* 2023) reported no direct association between acupuncture and QoL improvements but noted significant benefits in reducing pelvic, menstrual, and non-specific pelvic pain. However, the low quality of studies limits the strength of these conclusions. Furthermore, no association was found between QoL and Japanese-style acupuncture in the articles included in our review (Wayne PM *et al.* 2008). Beyond physical and psychological therapies, other complementary approaches have been explored. Regarding supplementation, curcumin, ginsenosides, and polyphenols have shown potential benefits for oxidative stress, inflammation, and pain modulation in endometriosis (Zaurito *et al.* 2024). It is, therefore, important to emphasize that herbal medicine is not without risks, and the conclusions of the review conducted by Zaurito *et al.* should not be interpreted as an endorsement of its systematic use. In fact, omega-3 and vitamin D supplementation have not been linked to QoL improvements (Abokhrais *et al.* 2020), nor has curcumin (Gudarzi *et al.* 2024). In addition, as demonstrated in a recent systematic review, manual therapy, cognitive behavioral therapy, and dietary interventions may also contribute to better QoL (Mazur-Bialy *et al.* 2024). These approaches are most effective when they minimize mental health side effects or additional pain, underscoring their suitability as part of a holistic care strategy. A meta-analysis (Del Pino-Sedeño *et al.* 2024) suggested that psychological interventions may improve pain and mental health in endometriosis patients; however, these findings should be interpreted with caution given the high risk of bias in the included studies. These findings collectively highlight the importance of a multidisciplinary approach, incorporating complementary therapies alongside (and not *in lieu* of) conventional care. Such integrated management may better address endometriosis’ physical, psychological, and social dimensions, providing a more comprehensive support to patients.

Consistent with these observations, endometriosis management requires a multidisciplinary approach involving specialists such as gynecologists, psychologists, and physiotherapists to address the physical, emotional, and social aspects of the disease. A systematic review by Fang *et al.* highlights the importance of multidisciplinary team care in managing pelvic pain and endometriosis, emphasizing patient needs for reliable information, validation of their experiences, respect for their preferences, and long-term treatment planning, including emotional and social support (Fang *et al.* 2024). Mick *et al.* emphasized the complexity of endometriosis and the need for individualized, long-term management (Mick *et al.* 2024). Ensuring equitable access to appropriate care and improving care coordination could significantly enhance patients’ QoL.

### Strengths and limitations

This systematic review study had several limitations. Many included studies had cross-sectional designs (*n* = 11/21), making it difficult to establish causal relationships or directions of the associations. These designs are prone to confounding variables and often have limited statistical power. Small sample sizes in some studies also reduced their power and generalizability.

Nevertheless, the rigorous methodology of this review, adhering to PRISMA guidelines and synthesizing evidence from lower-risk-of-bias studies only, ensures a robust approach. The exclusion of studies that did not report complete QoL scores reduced the number of included studies but ensured methodological consistency. However, the summary figures included results for factors associated with either individual QoL items or the overall QoL score (Fig. 3). This review comprehensively identified factors associated with QoL in women with endometriosis, regardless of their diagnostic method, aligning with the latest recommendations by the European Society of Human Reproduction and Embryology (ESHRE) that include non-surgical diagnoses (Becker *et al.* 2022). This study provides novel insights into socio-demographic characteristics, healthcare pathways, health status, and coping strategies compared with existing reviews. It confirms and expands current knowledge on mental health factors, endometriosis symptoms, and disease impact. While the findings are consistent with those of (Szypłowska *et al. *2023), this review provides a more detailed analysis of results based on the methodological quality of included studies. One important strength is the assessment of risk of bias for each study and the exclusion of studies with a high risk of bias, and the search of several databases.

This is the first systematic literature review to address these objectives in clinically- or surgically- diagnosed patients. It aims to inform management strategies focusing on non-clinical and non-surgical factors influencing QoL and improving outcomes for individuals with endometriosis. By highlighting current gaps and methodological limitations in the literature, this review provides a reference for future research and targeted interventions. 

### Conclusion and future perspectives

The studies published to date on non-pharmacological and non-surgical factors associated with QoL in women with endometriosis suggest that a better level of QoL is associated with more favorable socio-demographic characteristics (education, employment, income), a patient-centered care pathway, partner involvement, greater self-efficacy, physical activity, and certain non-drug interventions. In contrast, a low QoL was associated with poor health status, poor mental health, maladaptive coping strategies, unhealthy lifestyle (smoking and alcohol use), and high intensity of endometriosis pain and symptoms.

This systematic literature review highlights several gaps in current research. Few studies provide clear recommendations on the optimal duration and frequency of interventions aimed at improving QoL. While multidisciplinary care is crucial, limited research has explored how lifestyle habits influence QoL, warranting further longitudinal studies. Some studies reveal disparities in access to care based on socioeconomic level, but this issue remains underexplored globally (Fourquet *et al.* 2019, Westwood *et al.* 2023). In high-risk-of-bias studies, many observational investigations suffered from insufficient adjustment for confounding factors and lacked validated endometriosis diagnoses. While RCTs provide stronger evidence, they often face blinding challenges and high dropout rates.

Consensus on the QoL scales utilized is essential, with this review finding various instruments applied across studies, which could sometimes lead to inconsistent findings (e.g. age, BMI). While the SF-36 (generic) and EHP-30 (disease-specific) are the most common QoL scales used, their shorter versions (SF-12 and EHP-5) were also used to reduce participant burden (Bourdel *et al.* 2019, Nicolas-Boluda *et al.* 2021). Instruments such as WHOQOL and EQ-5D were less frequently utilized. This heterogeneity complicates cross-study comparisons and meta-analyses, emphasizing the need for standardized measures. In the context of endometriosis, it may be appropriate to recommend the use of the EHP-30 or its shorter version, the EHP-5, for disease-specific assessment, or alternatively to combine the EHP instruments with the SF-36 or SF-12 when broader comparisons with other conditions are warranted, as suggested by Bourdel *et al.*, who concluded that both the SF-36 and EHP-30 demonstrate good overall performance in endometriosis studies (Bourdel *et al.* 2019).

Key determinants of QoL remain underexplored, including patient characteristics (residence, central sensitization, neuropathic pain, treatment burden), reproductive and menstrual health (age at menarche, libido, parity, menopausal status), lifestyle (diet), and healthcare access (follow-up frequency, barriers to care). In addition, most studies identify cross-sectional associations without temporality information due to design limitations.

Despite the recognized importance of qualitative research in understanding the patient experience (Denny & Khan 2006), this review did not identify any qualitative studies. Publication bias may also be present, particularly as our review focused exclusively on peer-reviewed empirical studies and did not include gray literature, which can sometimes contain unpublished findings. This methodological choice was made to ensure a consistent level of scientific rigor across included studies. Nevertheless, no peer-reviewed studies examined factors associated with deterioration following non-pharmacological or non-surgical interventions – an area of clear clinical need. These gaps highlight the need for more robust evidence, including longitudinal, interventional, and qualitative research, to better inform clinical guidelines.

Integrating QoL measurement and its influencing factors into clinical consultations could enhance endometriosis management by enabling personalized care approaches. Effective strategies should encompass psychological support, patient education on self-management, and comprehensive care addressing the disease’s physical and mental health aspects. Social support and QoL assessment tools are also vital for improving overall well-being.

Given the low quality of the studies, further targeted research with more robust study designs is essential to deepen our understanding of the factors affecting QoL and to develop tailored, evidence-based interventions. Recognizing these factors is essential for optimizing care and enhancing patient well-being.

## Supplementary materials









## Declaration of interest

ZB’s PhD was partly funded by the French National Association for Research and Technology (ANRT) and Lyv Healthcare. MP and MA are Lyv Healthcare employees. Lyv Healthcare is the company that developed the digital program assessed in one of the studies included in this review (Breton *et al*. 2025). Other authors had no conflicts to declare. IARC disclaimer: where authors are identified as personnel of the International Agency for Research on Cancer/World Health Organization, the authors alone are responsible for the views expressed in this article and they do not necessarily represent the decisions, policy, or views of the International Agency for Research on Cancer/World Health Organization.

## Funding

ZB’s PhD was partly funded by the French National Association for Research and Technology (ANRT) and Lyv Healthcare.

## Author contribution statement

ZB and MK conceived and designed the study. ZB and GC performed the screening. ZB, GC, NM, MP, JC, and MA extracted data from the included studies. YMS and SN helped with study analysis. ZB drafted the original manuscript. ZB, GC, NM, YMS, MP, JC, SN, MA, and MK contributed to the interpretation of data discussed in the manuscript, revised the manuscript, and approved its final version.

## Data availability

The data underlying this article are available in the article and in its online supplementary material. The derived data generated in this research will be shared on reasonable request to the corresponding author.
