# Supplementary material for: Patient characteristics and lifestyle determinants of quality of life among women with endometriosis: a systematic review
Source: Reprod Fertil. 2026 May 20;7(2):RAF250094. doi: 10.1530/RAF-25-0094 (PMC13193072; doi:10.1530/RAF-25-0094)
Supplement: Supplementary file 1 [file supplementary_figure_1.pdf]

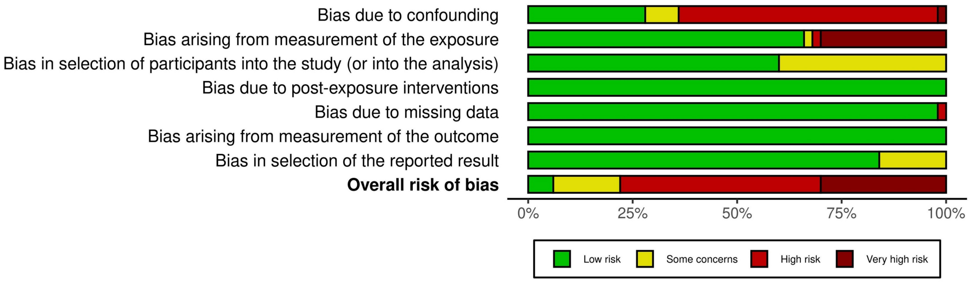

Bias due to confounding
Bias arising from measurement of the exposure
Bias in selection of participants into the study (or into the analysis)
Bias due to post-exposure interventions
Bias due to missing data
Bias arising from measurement of the outcome
Bias in selection of the reported result
Overall risk of bias
0%
25%
50%
75%
100%
Low risk
Some concerns
High risk
Very high risk

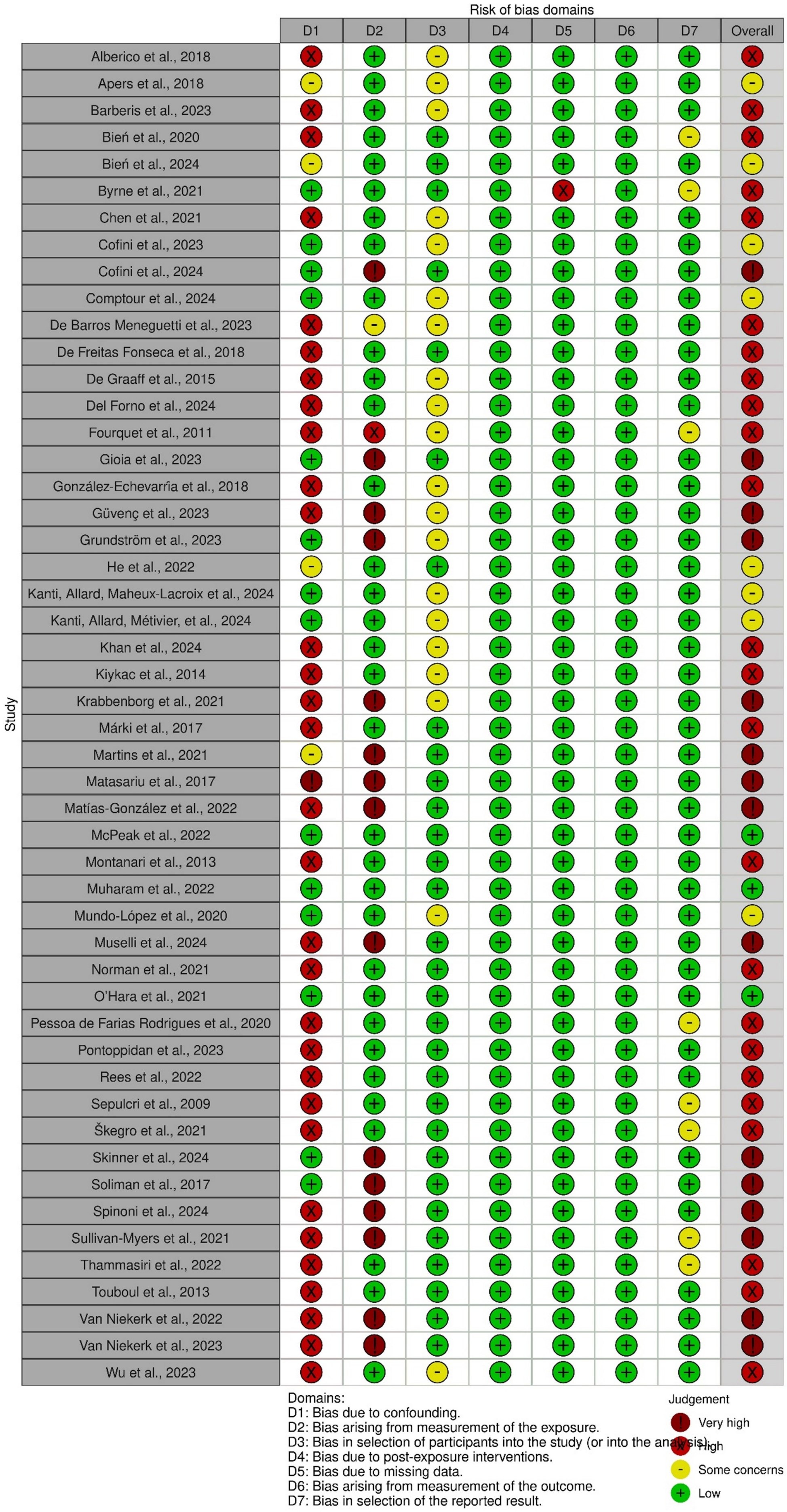

Risk of bias domains
D1
D2
D3
D4
D5
D6
D7
Overall
Study
Alberico et al., 2018
Apers et al., 2018
Barberis et al., 2023
Bień et al., 2020
Bień et al., 2024
Byrne et al., 2021
Chen et al., 2021
Cofini et al., 2023
Cofini et al., 2024
Comptour et al., 2024
De Barros Meneguetti et al., 2023
De Freitas Fonseca et al., 2018
De Graaff et al., 2015
Del Forno et al., 2024
Fourquet et al., 2011
Gioia et al., 2023
González-Echevarría et al., 2018
Güvenç et al., 2023
Grundström et al., 2023
He et al., 2022
Kanti, Allard, Maheux-Lacroix et al., 2024
Kanti, Allard, Métivier, et al., 2024
Khan et al., 2024
Kiykac et al., 2014
Krabbenborg et al., 2021
Márki et al., 2017
Martins et al., 2021
Matasariu et al., 2017
Matías-González et al., 2022
McPeak et al., 2022
Montanari et al., 2013
Muharam et al., 2022
Mundo-López et al., 2020
Muselli et al., 2024
Norman et al., 2021
O'Hara et al., 2021
Pessoa de Farias Rodrigues et al., 2020
Pontoppidan et al., 2023
Rees et al., 2022
Sepulcri et al., 2009
Škegro et al., 2021
Skinner et al., 2024
Soliman et al., 2017
Spinoni et al., 2024
Sullivan-Myers et al., 2021
Thammasiri et al., 2022
Touboul et al., 2013
Van Niekerk et al., 2022
Van Niekerk et al., 2023
Wu et al., 2023
Domains:
D1: Bias due to confounding.
D2: Bias arising from measurement of the exposure.
D3: Bias in selection of participants into the study (or into the analysis).
D4: Bias due to post-exposure interventions.
D5: Bias due to missing data.
D6: Bias arising from measurement of the outcome.
D7: Bias in selection of the reported result.
Judgement
Very high
High
Some concerns
Low
