# Supplementary material for: Patient characteristics and lifestyle determinants of quality of life among women with endometriosis: a systematic review
Source: Reprod Fertil. 2026 May 20;7(2):RAF250094. doi: 10.1530/RAF-25-0094 (PMC13193072; doi:10.1530/RAF-25-0094)
Supplement: Supplementary file 2 [file supplementary_figure_2.pdf]

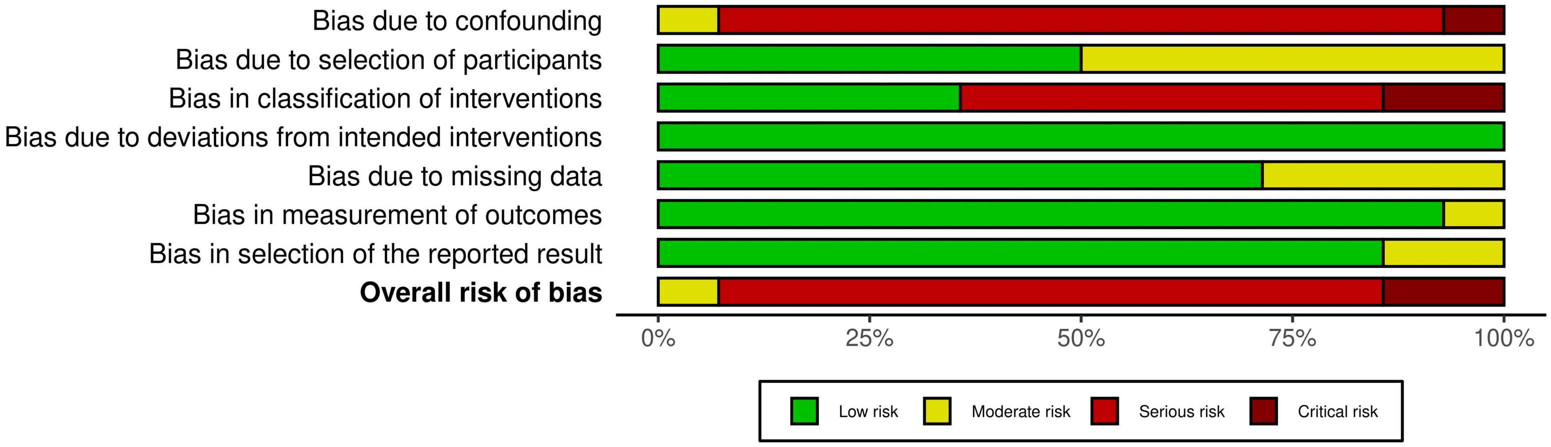
Bias due to confounding
Bias due to selection of participants
Bias in classification of interventions
Bias due to deviations from intended interventions
Bias due to missing data
Bias in measurement of outcomes
Bias in selection of the reported result
Overall risk of bias
0%
25%
50%
75%
100%
Low risk
Moderate risk
Serious risk
Critical risk

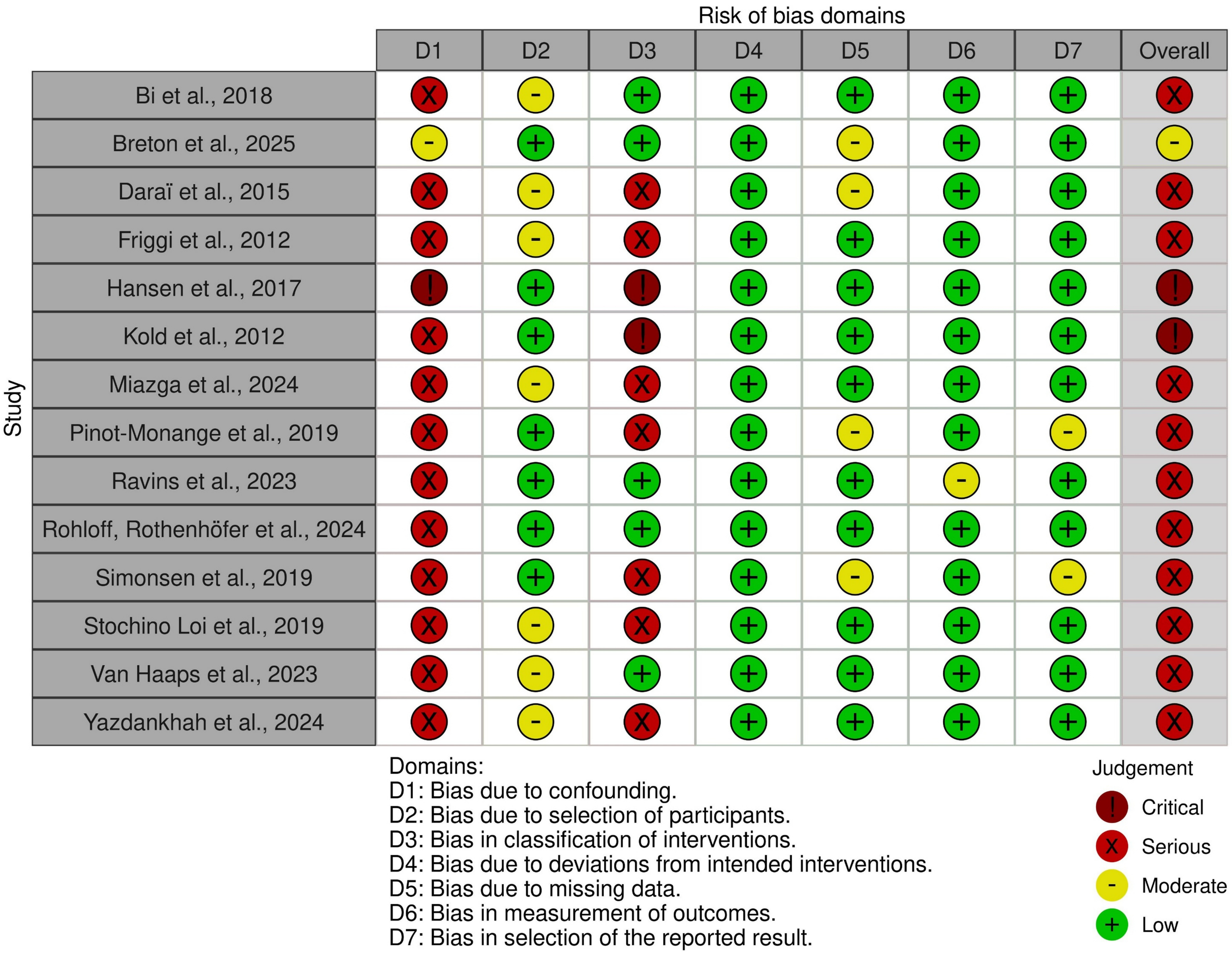
Risk of bias domains
D1
D2
D3
D4
D5
D6
D7
Overall
Study
Bi et al., 2018 X - + + + + + X
Breton et al., 2025 - + + + - + + -
Daraï et al., 2015 X - X + - + + X
Friggi et al., 2012 X - X + + + + X
Hansen et al., 2017 ! + ! + + + + !
Kold et al., 2012 X + ! + + + + !
Miazga et al., 2024 X - X + + + + X
Pinot-Monange et al., 2019 X + X + - + - X
Ravins et al., 2023 X + + + + - + X
Rohloff, Rothenhöfer et al., 2024 X + + + + + + X
Simonsen et al., 2019 X + X + - + - X
Stochino Loi et al., 2019 X - X + + + + X
Van Haaps et al., 2023 X - + + + + + X
Yazdankhah et al., 2024 X - X + + + + X
Domains:
D1: Bias due to confounding.
D2: Bias due to selection of participants.
D3: Bias in classification of interventions.
D4: Bias due to deviations from intended interventions.
D5: Bias due to missing data.
D6: Bias in measurement of outcomes.
D7: Bias in selection of the reported result.
Judgement
! Critical
X Serious
- Moderate
+ Low
