# Supplementary material for: Patient characteristics and lifestyle determinants of quality of life among women with endometriosis: a systematic review
Source: Reprod Fertil. 2026 May 20;7(2):RAF250094. doi: 10.1530/RAF-25-0094 (PMC13193072; doi:10.1530/RAF-25-0094)
Supplement: Supplementary file 3 [file supplementary_figure_3.pdf]

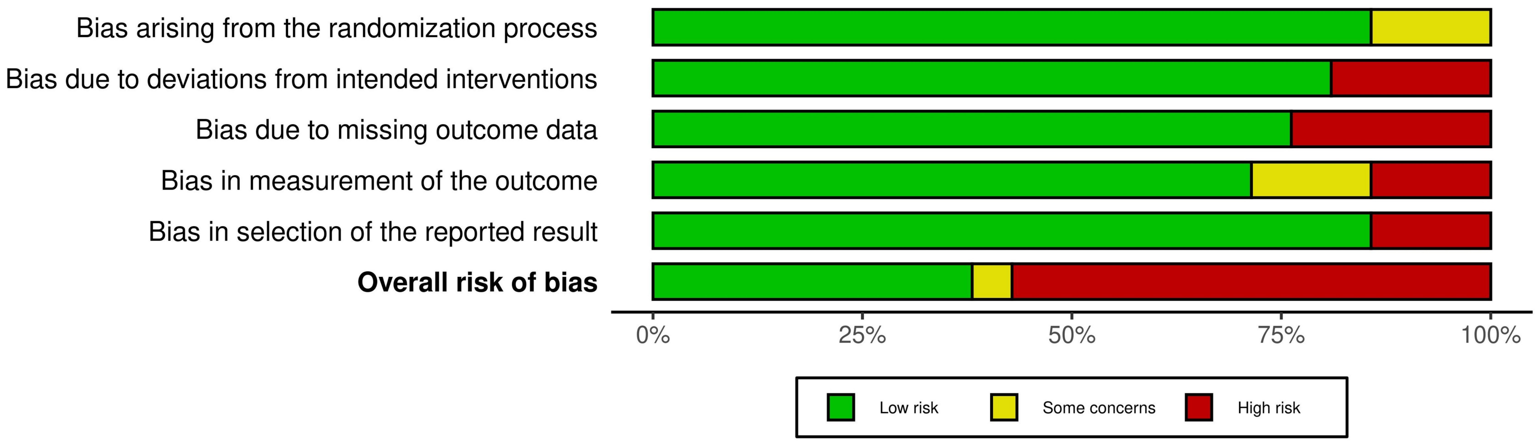
Bias arising from the randomization process
Bias due to deviations from intended interventions
Bias due to missing outcome data
Bias in measurement of the outcome
Bias in selection of the reported result
Overall risk of bias
0%
25%
50%
75%
100%
Low risk
Some concerns
High risk

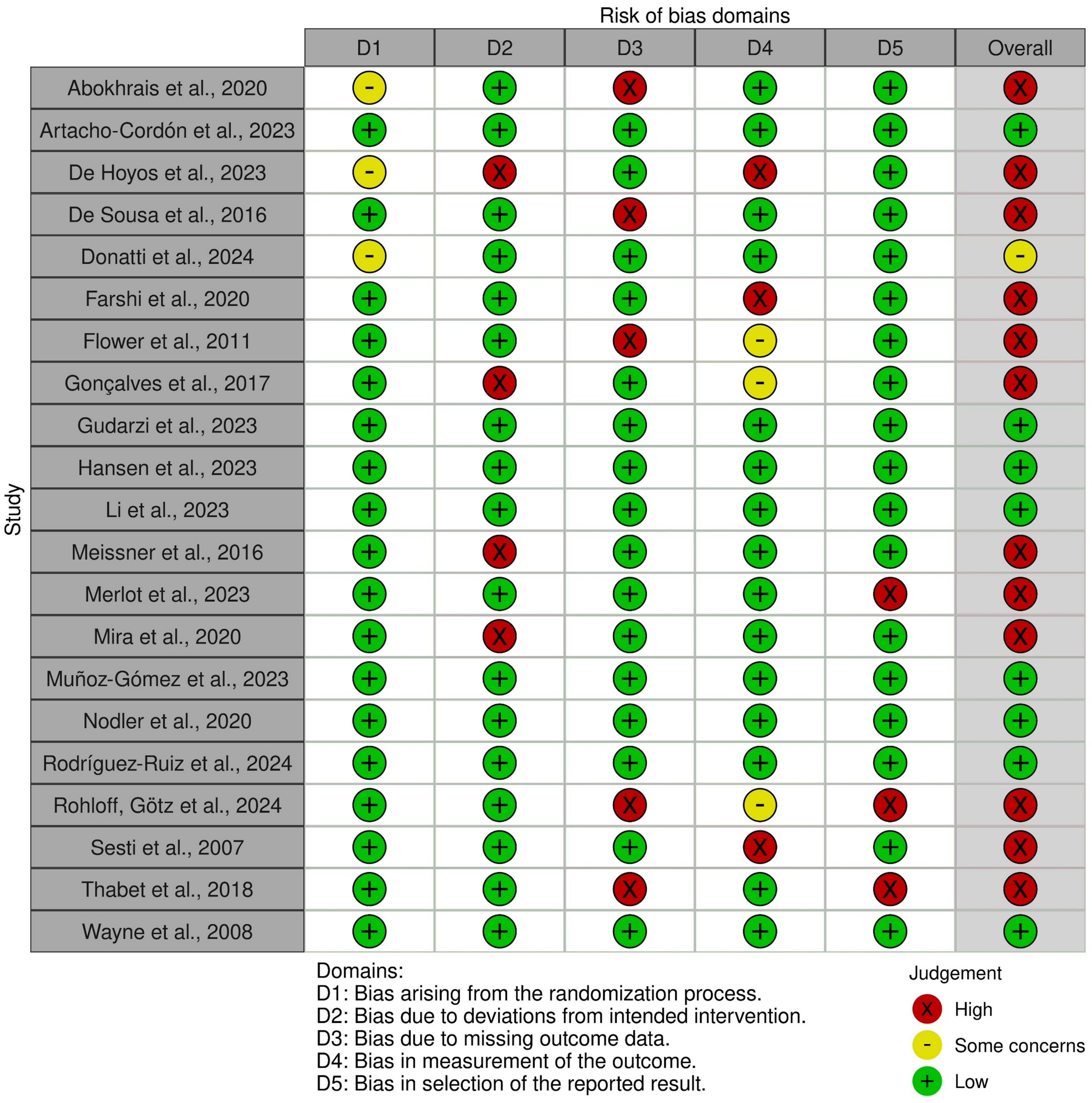
Risk of bias domains
D1 D2 D3 D4 D5 Overall
Study
Abokhrais et al., 2020 - + X + + X
Artacho-Cordón et al., 2023 + + + + + +
De Hoyos et al., 2023 - X + X + X
De Sousa et al., 2016 + + X + + X
Donatti et al., 2024 - + + + + -
Farshi et al., 2020 + + + X + X
Flower et al., 2011 + + X - + X
Gonçalves et al., 2017 + X + - + X
Gudarzi et al., 2023 + + + + + +
Hansen et al., 2023 + + + + + +
Li et al., 2023 + + + + + +
Meissner et al., 2016 + X + + + X
Merlot et al., 2023 + + + + X X
Mira et al., 2020 + X + + + X
Muñoz-Gómez et al., 2023 + + + + + +
Nodler et al., 2020 + + + + + +
Rodríguez-Ruiz et al., 2024 + + + + + +
Rohloff, Götz et al., 2024 + + X - X X
Sesti et al., 2007 + + + X + X
Thabet et al., 2018 + + X + X X
Wayne et al., 2008 + + + + + +
Domains:
D1: Bias arising from the randomization process.
D2: Bias due to deviations from intended intervention.
D3: Bias due to missing outcome data.
D4: Bias in measurement of the outcome.
D5: Bias in selection of the reported result.
Judgement
X High
- Some concerns
+ Low
