# Supplementary material for: Patient characteristics and lifestyle determinants of quality of life among women with endometriosis: a systematic review
Source: Reprod Fertil. 2026 May 20;7(2):RAF250094. doi: 10.1530/RAF-25-0094 (PMC13193072; doi:10.1530/RAF-25-0094)
Supplement: Supplementary file 4 [file supplementary_tables.pdf]

Supplementary Table 1: Justification for the impossibility of meta-analysis: studies categorized by quality of life scores, investigated factors, and statistical methods used

	Endometriosis-specific QoL score		QoL score non-specific to endometriosis		
Explored factors	**EHP-5**	**EHP-30**	**SF12**	**SF36**	**EQ-5D**
**Sociodemographic characteristics**					
Age		¤ McPeak 2018		¤ Cofini 2023<br>➢ Age as a continuous variable<br>➢ Association measured with coefficients β and p-value<br>¤ O'Hara 2021<br>➢ Age as a continuous variable<br>➢ Association measured with coefficients β and p-value<br>¤ Comptour 2024<br>➢ Age as a discrete variable<br>➢ Association measured with Relative Risk (RR) and p-value	
Education				¤ Cofini 2023<br>¤ O'Hara 2021	
Relationship status				¤ O'Hara 2021	
Financial status				¤ O'Hara 2021	
Occupational status				¤ Cofini 2023	
Health insurance				¤ O'Hara 2021	
**Healthcare pathway**					
Patient-centered endometriosis care		¤ Apers 2018			

Consultations with complementary medicine practitioners				¤ O'Hara 2021	
Quality of care				¤ Cofini 2023	
**Health status**					
Pregnancy				¤ Cofini 2023	
BMI		¤ Muharam 2022		¤ Cofini 2023	
Comorbidities				¤ Cofini 2023	
Stage of endometriosis				¤ Comptour 2024	
Poorer gastrointestinal QoL		¤ Mundo-López 2020			
Sleep quality		¤ Mundo-López 2020			
Endometrioma presence vs. other types of endometriosis		¤ Kanti, Allard, Maheux-Lacroix 2024			
Poorer sexual function		¤ Mundo-López 2020			
**Mental health**					
Anxiety level		¤ Mundo-López 2020	¤ He 2022		
Depression level		¤ Mundo-López 2020	¤ He 2022		
Anger / hostility		¤ Mundo-López 2020			
Presence of mental health issues		¤ Muharam 2022			
**Endometriosis symptoms**					
Pain symptoms severity (regrouped)		¤ Muharam 2022<br>➢ Pain as discrete variable with 2 modalities<br>➢ Associations measured with odds ratio (OR), CI and p-value		¤ O'Hara 2021	

		¤ Mundo-López 2020<br>➢ Pain as discrete variable with 3 modalities<br>➢ Associations measured with coefficient β, CI and p-value<br>¤ Kanti, Allard, Métivier 2024<br>➢ Pain as discrete variable with 2 modalities<br>➢ Associations measured with mean differences and p-value			
Dysmenorrhea level		¤ McPeak 2018			
Chronic pelvic pain level		¤ McPeak 2018			
Presence of abdominal wall pain		¤ McPeak 2018			
Higher back pain level		¤ McPeak 2018			
Fatigue		¤ Mundo-López 2020			
Duration of endometriosis-related symptoms		¤ Kanti, Allard, Maheux-Lacroix 2024			
**Endometriosis impact**					
Active involvement of partner				¤ O'Hara 2021	
**Lifestyle**					
Smoking				¤ Cofini 2023	
Physical activity				¤ Cofini 2023	

Alcohol				¤ Cofini 2023	
**Coping strategies**					
Pain catastrophizing		¤ McPeak 2018			
Number of self-care strategies used	¤ Wu 2024	¤ Norman 2021		¤ O'Hara 2021	
Self-efficacy				¤ O'Hara 2021	
Chronic disease management plan				¤ O'Hara 2021	
Social support		¤ Mundo-López 2020			
**Interventions**					
Omega 3 dietary supplement			¤ Nodler 2020		
Vitamin D dietery supplement			¤ Nodler 2020		
Acupuncture		¤ Li 2023<br>¤ Wayne 2008			
Psychological intervention		¤ Hansen 2023			
Exercise program		¤ Artacho-Cordón			
Digital health program	¤ Breton 2025				¤ Breton 2025
Cognitive behavioral therapy				¤ Donatti 2024	
Curcumin		¤ Gudarzi 2024			
Specific mindfulness- and acceptance-based psychological intervention (MY-ENDO)		¤ Hansen 2023			
Integral HAMMAM experience		¤ Rodríguez-Ruiz 2024			
Manual therapy protocol		¤ Muñoz-Gómez 2023		¤ Muñoz-Gómez 2023	

This table categorizes studies based on the factors potentially associated with quality of life and the quality of life score used. When at least three studies examined the same factor using the same score, the statistical methodologies employed were detailed.

Abbrevations: β (Beta): Regression Coefficient; BMI: Body Mass Index; CI: Confidence Interval; EHP-5: Endometriosis Health Profile – 5 items; EHP-30: Endometriosis Health Profile – 30 items; EQ-5D: EuroQol 5 Dimensions; OR: Odds ratio; RR: Relative Risk; SF-12: 12-Item Short Form Health Survey; SF-36: 36-Item Short Form Health Survey

Supplementary Table 2: Summary of studies with high risk of bias

**Author, year, country**	**Design / Purpose**	**Intervention or exposure potentially associated with QoL / QoL score and data point**	**Participants (n, age)**	**Level of quality of life at baseline**	**Factors significantly associated with improved quality of life**	**Factors significantly associated with a deterioration in quality of life**
Abokhrais et al., 2020 UK	RCT<br><br>Evaluate the efficacy of Omega-3 polyunsaturated fatty acids for endometriosis-associated pain.	Intervention: Omega 3 PUFA supplementation 1000mg twice a day<br><br>Control: 1000mg olive oil capsule twice a day<br><br>SF-12<br>Baseline | 8 weeks	Intervention:<br>n=14<br>Mean age = 35.43<br><br>Control:<br>n=13<br>Mean age = 36.08	Mean (SD)<br><br>Intervention:<br>Physical component = 2.58 (0.59)<br>Mental component= 2.94 (0.63)<br><br>Control:<br>Physical component = 2.65 (0.76)<br>Mental component = 2.79 (0.67)	No significant results	No significant results
Alberico et al., 2018 Italy	Retrospective study<br><br>Explore the potential benefits of pregnancy on endometriosis symptoms, mental health and QoL.	Exposure:<br>- Pregnancy (dichotomous)<br>- Pain symptoms: dysmenorrhea, dyspareunia, non-menstrual pain, dyschezia (NRS)<br><br>Control: None<br><br>SF-12	n=131<br><br>Mean age (SD) = 36 (0)	Mean (SD)<br><br>Physical component: 36.4 (8.5)<br><br>Mental component: 41.5 (8.2)	**Pregnancy** (mean change): p<0.001 for physical (42.5 post vs. 36.4 pre) and mental (47.1 vs. 41.5) component<br><br>**Improvement in pain symptoms** (correlation):<br>- **Dysmenorrhea**:<br>Physical: r=-0.44 p=<0.001<br>Mental: r=-0.29 p=0.001<br>- **Dyspareunia:**<br>Physical: r=-0.42 p=<0.001	None

Author, year, country	Design / Purpose	Intervention or exposure potentially associated with QoL / QoL score and data point	Participants (n, age)	Level of quality of life at baseline	Factors significantly associated with improved quality of life	Factors significantly associated with a deterioration in quality of life
		Before pregnancy | Two years after delivery			Mental: r=-0.32 p=0.002<br>**- Non menstrual pain:**<br>Physical: r=-0.35 p=0.004<br>Mental: r=-0.27 p=0.027<br>**- Dyschezia:**<br>Physical: r=-0.32 p=0.015	
Barberis et al., 2023 Italy	Cross sectional<br><br>Test the hypothesis that the association between trait emotional intelligence (EI) and QoL in individuals with endometriosis is mediated by illness perceptions, while also examining the relationships with general distress (depression, anxiety, and stress).	Exposure: (continuous)<br>- Illness Perception (IP)<br>- General Distress (GD)<br>- Emotional Intelligence (EI)<br><br>Control: None<br><br>EHP-30<br>One data point	n=364<br><br>Mean age (SD) = 33.87 (8.64)	Mean (SD) = 2.48 (0.79)	**Higher trait Emotional Intelligence:**<br>correlation: r=-0.43 p<0.1<br>mediation model B(CI):<br>EI -> IP -> GD -> QoL = -0.06 (-0.08;-0.03)<br>EI -> IP -> QoL = -0.21 (-0.25;-0.11)<br>EI -> GD -> QoL = -0.23 (-0.27;-0.13)	**General Distress:**<br>correlation: r=0.67 p<0.1<br>mediation model B(CI):<br>GD -> QoL = 0.44 (0.37;0.69)<br><br>**Poor Illness Perception:**<br>correlation: r=0.67 p<0.1<br>mediation model B(CI):<br>IP -> QoL = 0.55 (0.12;0.22)<br>IP -> GD -> QoL = 0.16 (0.03;0.07)
Bi et al., 2018 China	Retrospective study<br><br>Evaluate the effect of neuromuscular electrical stimulation (NMES) for the	Intervention: neuromuscular electrical stimulation (NMES), 30 minutes each session, once daily, 3 sessions weekly for a total of 10 weeks (dichotomous)	Intervention:<br>n=83<br>Mean age (SD) = 31.6 (3.6)<br><br>Control:<br>n=71<br>Mean age (SD) = 32.2 (4.1)	Mean (SD)<br><br>Intervention:<br>Physical: 52.1 (13.3)<br>Mental: 57.6 (12.8)<br><br>Control:<br>Physical: 52.4 (12.1)<br>Mental: 59.1 (13.5)	**NMES after 10-week treatment**, MD (CI)<br>intervention group vs. control:<br>Physical: 7.8 (6.3;8.9), p<0.01<br>Mental: 7.3 (6.1;8.5), p<0.01	None

Author, year, country	Design / Purpose	Intervention or exposure potentially associated with QoL / QoL score and data point	Participants (n, age)	Level of quality of life at baseline	Factors significantly associated with improved quality of life	Factors significantly associated with a deterioration in quality of life
	treatment of endometriosis associated pain (EAP).	Control: waiting list<br><br>SF-36<br>Baseline | 5 weeks | 10 weeks				
Bień et al., 2020 Poland	Cross sectional<br><br>Assess QoL and identify and analyse its determinants in women with endometriosis.	Exposure:<br>- Age (continuous)<br>- Education (dichotomous)<br>- Relationship status (dichotomous)<br>- Perceived family wealth (3 categories)<br>- BMI (4 categories)<br>- Is endometriosis treatment expensive (dichotomous)<br>- Impact of the condition on the relationship with the partner (dichotomous)<br>- Symptoms of the condition disrupt day-to-day functioning (dichotomous)<br>- Pain: dyspareunia, painful periods (dichotomous)<br><br>Control: None<br><br>WHOQOL-BREF<br>One data point	n = 309<br><br>Mean age (SD) = 30.86 (5.57)	Mean (SD)<br><br>Overall: 3.30 (0.91)<br><br>Psychological: 13.33 (2.28)<br>Physical: 11.52 (3.02)<br>Social: 12.90 (3.51)<br>Environment: 12.85 (2.68)	**Higher acceptance of illness:**<br>Physical: β=0.316, p=0.000<br>Psychological: β=0.348, p=0.000<br>Social: β=0.298, p=0.003<br>Environmental: β=0.259, p=0.001<br><br>**Higher education:**<br>Physical: β=0.099, p=0.044	**BMI - obesity:**<br>Psychological: β=-0.249, p=0.000<br>Environmental: β=-0.170, p=0.005<br><br>**Negative impact of symptoms on the relationship with partner:**<br>Physical: β=0.159, p=0.005<br>Social: β=0.223, p=0.009<br><br>**Dyspareunia:**<br>Psychological: β=0.168, p=0.020<br>Social: β=0.225, p=0.003<br><br>**Pain intensity:**<br>Physical: β=-0.322, p=0.000<br><br>**Pain experienced during menstrual periods disrupt day-to-day functioning:**<br>Psychological: β=0.174, p=0.012<br><br>**Cost of endometriosis treatment:**<br>Environmental: β=0.130, p=0.007

Author, year, country	Design / Purpose	Intervention or exposure potentially associated with QoL / QoL score and data point	Participants (n, age)	Level of quality of life at baseline	Factors significantly associated with improved quality of life	Factors significantly associated with a deterioration in quality of life
						**Poor perceived family wealth:**<br>Environmental: β=0.123, p=0.029<br><br>**Age:**<br>Social: β=-0.197, p=0.011
Byrne et al., 2021 UK	Cohort study<br><br>Investigate factors that influence QoL after laparoscopic excision of deep rectovaginal endometriosis.	Exposure:<br>- Age (+/- 45yo)<br>- Smoking status (dichotomous)<br>- BMI (2 categories)<br><br>Control: None<br><br>EQ-VAS<br>Baseline | 6 months | 24 months after surgery	n=8,368 at baseline<br>n=5,260 at 6 months<br>n=2,115 at 24 months<br><br>Mean age (SD) = NA	Mean pre-operative score: 55.1/100 (95 % CI 54.6–55.6)	None (only post operatively)	**Pre-op BMI >35:** (MD = 69.4, 95%CI = 66.8–71.9), compared to slimmer women (72.2, 71.5–72.9; P = 0.014)<br><br>**Pre-op smoker:** (6.9 points, p<0.01)
Chen et al., 2021 Italy China	Cross sectional<br><br>Evaluate the difference between Chinese and Italian populations with endometriosis in terms of symptoms, comorbidities, and QoL.	Exposure:<br>Ethnicity (Chinese / Italian)<br><br>Control: None<br><br>SF-12<br>One data point	Italian:<br>n=196<br>Mean age (SD) = 36.3 (6.4)<br><br>Chinese:<br>n=175<br>Mean age (SD) = 36.5 (6.7)	Mean (SD)<br><br>Physical score:<br>Chinese = 46.5 (8.7)<br>Italian = 41.5 (9.9)<br><br>Mental score<br>Chinese = 47.1 (8.8)<br>Italian = 37.2 (10.7)	**Chinese ethnicity vs. Italian** (MD, SD):<br>Physical: 46.5 (8.7) vs 41.5 (9.9), p < 0.0001<br>Mental: 47.1 (8.8) vs 37.2 (10.7), p < 0.0001	None
Cofini et al., 2024 Italy	Cross sectional<br><br>Characterize the clinical and sociodemograph	Exposure: Women with endometriosis and chronic pelvic pain (CPP)	Exposure:<br>n=661<br>Mean age (SD) = 34.7 (7.8)	Mean (SD)<br><br>Exposure:<br>Physical: 35.27 (9.48)<br>Mental: 32.96 (10.50)	None	**Chronic pelvic pain**<br><br>Mean difference p-value exposure vs. control:<br>Physical: <0.001

Author, year, country	Design / Purpose	Intervention or exposure potentially associated with QoL / QoL score and data point	Participants (n, age)	Level of quality of life at baseline	Factors significantly associated with improved quality of life	Factors significantly associated with a deterioration in quality of life
	ic characteristics, lifestyles, quality of life, and perceptions of quality of care in women with endometriosis who reported chronic pelvic pain during the Covid pandemic.	Control: Women with endometriosis without CPP<br><br>SF-36<br>One data point	Control:<br>n=384<br>Mean age (SD) = 35.2 (7.9)	Control:<br>Physical: 44.88 (9.55)<br>Mental: 37.06 (11.74)		Mental: <0.001<br>OR (95%CI) p-value:<br>Physical: 0.92 (0.90-0.94) <0.001<br>Mental: 0.97 (0.96-0.99) 0.001
Daraï et al., 2015 France	Interventional<br><br>Evaluate the potential role of osteopathic manipulative therapy (OMT) on QOL of patients with Deep Infiltrating Endometriosis (DIE) and colorectal involvement.	Intervention: osteopathic manipulative therapy (OMT), one session, median 60 minutes, range 45-73 minutes<br><br>Control: None<br><br>SF-36<br>Before and after intervention. The median time between questionnaires was 24 days (range: 15–53)	n=15<br><br>Median age (range) = 30.4 (22-39)	Mean (SD)<br><br>Physical: 41.4 (19.2)<br>Mental: 31.5 (17.5)	**Osteopathic manipulative therapy (OMT):** MD (SD)<br>Physical: 56.2 (20.9) p=0.03<br>Mental: 54.5 (21.6) p=0.0009	None
De Barros Meneguetti et al., 2023 Brazil	Cross sectional<br><br>Evaluate QoL, anxiety, and depression in women with endometriosis, and to correlate these parameters with	Exposure:<br>- Pain (VAS +/- 7/10)<br>- Anxiety (BAI - continuous)<br>- Depression (BDI - continuous)<br><br>Control: None<br><br>EHP-30	Severe pain:<br>n=62<br>Mean age (SD) =33.89 (5.71)<br><br>Mild/moderate pain:<br>n=40<br>Mean age (SD) =<br>Severe pain: 33.85	Mean (SD):<br><br>Severe pain: 48.88 (16.02)<br><br>Mild/moderate: 23.32 (15.93)	**Low depression level** (correlation)**:**<br>severe pain: p<0.001, r=0.54<br>mild/moderate pain: p=0.0063, r=0.43<br><br>**Low anxiety level** (correlation)**:**<br>severe pain: p<0.001, r=0.56<br>mild/moderate pain:	**High pain level** (mean, SD)**:** 48.88 (16.02) vs. 23.32 (15.93), p < 0.001

**Author, year, country**	**Design / Purpose**	**Intervention or exposure potentially associated with QoL / QoL score and data point**	**Participants (n, age)**	**Level of quality of life at baseline**	**Factors significantly associated with improved quality of life**	**Factors significantly associated with a deterioration in quality of life**
	pain intensity.	One data point	(5.60)		p=0.0095, r=0.41	
De Freitas Fonseca et al., 2018 Brazil	Cross sectional<br><br>Assess the correlation between different pain symptoms and different domains of women's HRQoL.	Exposure:<br>Age (continuous)<br>Dysmenorrhea (NRS)<br>Deep dyspareunia (NRS)<br>CPP (NRS)<br>Dyschezia menstrual (NRS)<br>Dyschezia non menstrual (NRS)<br><br>Control: None<br><br>SF-36 | EHP-30<br>One data point	n=77<br><br>Median age (range) = 35 (21-46)	No precise data	None	**Dysmenorrhea**<br><br>**CPP**<br><br>Correlated with all domains of the SF36 and the EHP30 (core instrument) questionnaires (P<0.05)
De Graaff et al., 2015 Netherlands	Retrospective study<br><br>Assess the extent to which outcome measures in endometriosis-related QoL studies are influenced by the setting of patient recruitment.	Exposure:<br>Recruitment setting (Tertiary care center for endometriosis; Secondary care centers; Endometriosis patient association)<br><br>Control: None<br><br>SF-36<br>One data point	Tertiary care center for endometriosis:<br>n=135<br>Median age (range) = 36 (22-55)<br><br>Secondary care centers:<br>n=63<br>Median age (range) = 37 (23-59)<br><br>Endometriosis patient association:<br>n=291<br>Median age (range) = 35 (20-	Mean<br><br>Tertiary center:<br>Physical: 46.2<br>Mental: 47.2<br><br>Tertiary center:<br>Physical: 50.4<br>Mental: 50.0<br><br>Association:<br>Physical: 45.0<br>Mental:47.2	None	Median difference<br><br>**Tertiary care vs. Secondary care**<br>Physical: 46.2 vs. 50.4<br>Mental: 46.2 vs.49.6<br>p=0.001<br><br>**Patient association vs. Secondary care**<br>Physical: 45.0 vs. 50.4<br>Mental: 44.6 vs.49.6<br>p=0.018

Author, year, country	Design / Purpose	Intervention or exposure potentially associated with QoL / QoL score and data point	Participants (n, age)	Level of quality of life at baseline	Factors significantly associated with improved quality of life	Factors significantly associated with a deterioration in quality of life
			58)			
De Hoyos et al., 2023 Puerto Rico	RCT<br><br>Adapt and test the efficacy of an Environmental Enrichment intervention on pelvic pain, mental health, perceived stress, quality of life, and systemic inflammation in endometriosis patients.	Intervention:<br>- Environmental Enrichment interventions: multi-modal psychosocial intervention consisting of increased social interaction, novelty, and open spaces<br><br>Control group:<br>Waiting list, education only<br><br>EHP-30<br>Baseline | six weeks | 3-months post intervention	Intervention:<br>n=29<br>Mean age = 32.7<br><br>Control:<br>n=27<br>Mean age = 34.0	Approximative mean:<br>Intervention: 60<br>Control: 60	**Environmental Enrichment interventions**<br><br>Mean difference between baseline and follow-up for intervention group: p=0.006. But no statistically significant difference compared to control group.	None
De Sousa et al., 2016 Brazil	RCT<br><br>Observe the effects of an acupuncture protocol on CPP, dyspareunia, and QoL in women with endometriosis.	Intervention:<br>Acupuncture - 5 treatments sessions, 1 per week, 40 minutes<br><br>Control: Placebo, needles inserted 3 cm apart from the points of energy<br><br>EHP-30<br>Pre-therapy | Post-therapy | 2 months after therapy	Intervention:<br>n=20<br>Mean age (SD) = 30.45 (5.89)<br><br>Control:<br>n=22<br>Mean age (SD) = 31.14 (6.92)	Mean (SD)<br><br>Intervention:71.50 (16.31)<br><br>Control: 66.36 (14.65)	**Acupuncture treatment** in all domains except infertility - MD (SD):<br>Intervention = 31.0 (10.21)<br>Control = 56.59 (11.89)<br>p<0.001	None
Del Forno et al., 2024 Italy	Cross-sectional<br><br>Assess the	Exposure:<br>- presence of dyspareunia	n=334<br><br>Isolated	Mean (SD)<br><br>Physical wellbeing: 86.9	None	**Concomitant dyspareunia** (correlation): p<0.001

Author, year, country	Design / Purpose	Intervention or exposure potentially associated with QoL / QoL score and data point	Participants (n, age)	Level of quality of life at baseline	Factors significantly associated with improved quality of life	Factors significantly associated with a deterioration in quality of life
	temporal relation between deep and superficial dyspareunia in women reporting both symptoms (concomitant dyspareunia) and the impact on quality of life (QoL).	(dichotomous)<br>- type of dyspareunia (categories)<br><br>Control:<br>- women with isolated dyspareunia or no dyspareunia<br><br>SF-36<br>One data point	superficial dyspareunia n=21<br>Isolated deep dyspareunia n=87<br>Concomitant dyspareunia n=145<br>No dyspareunia n=81<br><br>Age n, (%)<br><=35yo: 93 (27.8)<br>36-40yo: 88 (26.3)<br>41-45yo: 76 (22.8)<br>>45yo: 77 (23.1)	(20.4)<br>Physical role limitations: 68.3 (38.2)<br>Bodily Pain: 63.7 (27.7)<br>General: 54.9 (21.2)<br>Fatigue/Vitality: 49.0 (18.2)<br>Social: 61.1 (26.1)<br>Emotional role limitations: 61.1 (42.9)<br>Emotional well-being: 57.9 (17.2)		
Farshi et al., 2020 Iran	RCT<br><br>Determine the effects of selfcare counselling on depression and anxiety and on QoL among women with endometriosis.	Intervention: Self-care counselling - Seven sessions on a weekly basis, 60-90 minutes<br><br>Control: Received routine care<br><br>SF-36<br>Before | 4 weeks after the intervention	Intervention:<br>n=38<br>Mean age (SD) =34.8 (6.0)<br><br>Control:<br>n=38<br>Mean age (SD) =34.0 (6.2)	Mean (SD)<br><br>Intervention:<br>Physical: 55.0 (8.1)<br>Mental: 53.3 (6.2)<br><br>Control:<br>Physical: 43.8 (6.6)<br>Mental: 47.5 (7.5)	**Counselling group:**<br>Physical health: MD = 17.2, 95%CI: 13.8 to 20.5, p<0.001<br>Mental health: MD = 12.0, 95%CI: 9.0 to 14.9, p<0.001	None
Flower et al., 2011 UK	RCT<br><br>Test the feasibility of a novel methodology for investigating individualized decoctions of	Intervention: Chinese Herbal Medicine (CHM), individualized decoction, 16-week, formulations of between 10 and 15 herbs, with a daily dosage amounting 150g-250g	Intervention:<br>n=13<br>Mean age (SD) =33.2 (7.2)<br><br>Control:<br>n=15<br>Mean age (SD) = 35.7 (8)	No data	None	None

Author, year, country	Design / Purpose	Intervention or exposure potentially associated with QoL / QoL score and data point	Participants (n, age)	Level of quality of life at baseline	Factors significantly associated with improved quality of life	Factors significantly associated with a deterioration in quality of life
	Chinese Herbal Medicine (CHM) rigorously and to gather preliminary data on the treatment effect of CHM for a larger definitive trial.	Control: Waiting list, therapeutically inert placebo decoction<br><br>EHP-30<br>Baseline | 16 weeks				
Fourquet et al., 2011 Puerto Rico	Cross sectional<br><br>Quantify the impact of endometriosis-related symptoms on physical and mental health status, HRQoL, and work-related aspects (absenteeism, presenteeism, work productivity, activity impairment).	Exposure: Symptoms (item of SF-12 and EHP-5 score)<br><br>Control: None<br><br>SF-12 | EHP-5<br>One data point	n=193<br><br>Median age (range) = 34.0 (18-52)	Mean (SD)<br><br>SF-12:<br>Physical: 38.4 (6.7)<br>Mental: 39.5 (6.2)<br><br>EHP-5: no data	None	None
Friggi et al., 2012 Brazil	Interventional<br><br>Evaluate the effectiveness of submitting women with endometriosis and CPP to a therapeutic	Intervention: Physical therapy and psychological intervention: 2,5h sessions, once a week for 10 weeks<br><br>Control: None	n=26<br><br>Mean age (SD) = 32.2 (1.3)	Mean (SD)<br><br>Role physical: 61 (3.7)<br>Bodily pain: 33 (3.9)<br>General health: 43 (4.0)<br>Social functioning: 45 (5.0)<br>Role emotional: 33 (7.4)<br>Mental health: 37 (4.4)<br>Vitality: 30 (3.9)	MD (SD)<br><br>**Intervention with physical therapy and psychological intervention** (only for physical function and vitality item):<br>Vitality: 30 (3.9) vs. 39 (4.6) $p<0.05$<br>Physical functioning: 26 (6.5)	None

Author, year, country	Design / Purpose	Intervention or exposure potentially associated with QoL / QoL score and data point	Participants (n, age)	Level of quality of life at baseline	Factors significantly associated with improved quality of life	Factors significantly associated with a deterioration in quality of life
	protocol involving physical and psychological therapy.	SF-36<br>Baseline | 10 weeks		Physical functioning: 26 (6.5)	vs. 38 (7.5) p<0.05	
Gioia et al., 2023 Italy	Cross sectional<br><br>Exploring the still understudied relationship between alexithymia and HRQoL in endometriosis conditions, evaluating the mediating role of perceived loneliness.	Exposure:<br>- Alexithymia (the inability to recognize or describe one's own emotions)<br>- Loneliness<br><br>Control: None<br><br>EHP-30<br>One data point	n=435<br>Mean age (SD)=35.75 (6.72)	Mean (SD)<br><br>Pain: 49 (4.24)<br>Control and Powerlessness: 24.5 (0.71)<br>Emotional Well-Being: 27.5 (0.71)<br>Social Support: 16.5 (0.71)<br>Self-Image: 13.5 (2.12)	None	Correlation p<br><br>**Loneliness**: r=0.479 p<0.001
Gonçalves et al., 2017 Brazil	RCT<br><br>Evaluate the effect of yoga on endometriosis-related pain.	Intervention: Yoga program twice a week for 2 months<br><br>Control: Endometriosis treated with medication and/or one individual physical therapy session a week.<br><br>EHP-30<br>Baseline | Upon completion of the yoga program	Intervention:<br>n=28<br>Mean age (SD) = 34.5 (7.4)<br><br>Control:<br>n=12<br>Mean age (SD) = 35.75 (4.7)	Mean (SD)<br><br>Intervention:<br>Pain: 60.80 (15.59)<br>Control and powerlessness: 68.90 (18.13)<br>Emotional well-being: 65.03 (21.41)<br>Social support: 66.07 (24.62)<br>Self-image: 66.37 (26.40)<br><br>Control:<br>Pain: 58.71 (15.41)<br>Control and powerlessness: 66.67 (20.41)<br>Emotional well-being: 69.44	**Yoga** (in all domains of EHP-30 except social support - mean (SD))<br><br>Pain<br>Exposure 32.39 (21.95), control 55.05 (21.49)<br>Control and powerlessness<br>Exposure 34.44 (15.71), control 54.17 (16.67)<br>Emotional well-being<br>Exposure 41.67 (15.67), control 48.15 (29.47)<br>Self-image<br>Exposure 41.11 (25.68), control 43.52 (33.28)	None

Author, year, country	Design / Purpose	Intervention or exposure potentially associated with QoL / QoL score and data point	Participants (n, age)	Level of quality of life at baseline	Factors significantly associated with improved quality of life	Factors significantly associated with a deterioration in quality of life
				(24.51)<br>Social support: 59.38 (23.16)<br>Self-image: 52.08 (32.59)		
González-Echevarría et al., 2019 Puerto Rico	Cross sectional<br><br>Evaluate the effect of coping strategies on endometriosis.	Exposure: Coping strategies<br><br>Control: None<br><br>EHP-5<br>One data point	n=24<br><br>Age: 9 participants between 13-19; 15 participants between 20-25	n(%) never/low/high<br><br>Work and/or Study:<br>3 (12.5%) / 9 (37.5%) / 12 (50.0%)<br><br>Walking:<br>1 (4.2%) / 13 (54.2%) / 10 (41.7%)<br><br>The Pain Controls Your Life:<br>1 (4.2%) / 12 (50.0%) / 11 (45.8%)<br><br>Mood Changes:<br>2 (8.3%) / 10 (41.7%) / 12 (50.0%)<br><br>Others Do Not Understand You:<br>5 (20.8%) / 8 (33.3%) / 11 (45.8%)<br><br>Physical Changes<br>7 (29.2%) 8 (33.3%) 9 (37.5%)	Correlation<br><br>**Cognitive restructuring**<br>work and study −0.570 p<0.01, can't walk −0.471 p<0.05, activity impairment −0.497 p<0.05, mood changes −0.411 p<0.05<br><br>**Emotional expression**<br>work and study −0.410 p<0.05<br><br>**Social support**<br>work and study −0.411 p<0.05, can't walk 0.550 p<0.01	Correlation<br><br>**Auto-criticism** (all domains except can't walk)<br>work and study 0.511 p<0.05, activity impairment 0.521 p<0.01, mood changes 0.441 p<0.05, no one understands 0.477 p<0.05, physical changes 0.433 p<0.05<br><br>**Social withdrawal**<br>Social withdrawal work and study 0.466 p<0.05, mood changes 0.467 p<0.05, no one understands 0.449 p<0.05, physical changes 0.424 p<0.05
Grundström et al., 2023 Sweden	Cross sectional<br><br>Explore experiences of validating and invalidating communication	Exposure:<br>Levels of validation and invalidation from healthcare providers, employers, and family/friends	n=427<br>Mean age (SD)=34 (8.45)	Mean (SD): 57.08 (17.67)	Correlation p<br><br>**High levels of validation and low levels of invalidation from:**<br>**- healthcare providers:** -0.36 p<0.001	None

Author, year, country	Design / Purpose	Intervention or exposure potentially associated with QoL / QoL score and data point	Participants (n, age)	Level of quality of life at baseline	Factors significantly associated with improved quality of life	Factors significantly associated with a deterioration in quality of life
	in three contexts (with healthcare providers, employers, and family/friends), and whether this may predict health related quality of life (HRQoL) in women with endometriosis.	Control: None<br><br>EHP-30<br>One data point			**- close family and friends:** -0.34 p<0.001<br><br>B p<br>- healthcare providers: -0.15 p<0.001<br>- close family and friends: -0.11 p=0.007	
Güvenç et al., 2023 Turkey	Cross sectional<br><br>Examine the HRQoL of Turkish women with endometriosis within the framework of the Attachment-Diathesis Model of Chronic Pain.	Exposure:<br>- Pain (PCS)<br>- Coping strategies (WCI)<br><br>Control: None<br><br>EHP-30<br>One data point	n=174<br><br>Mean age (SD) = 30.86 (6.19)	No data	**Perceived controllability** β=0.323 p<0.01<br><br>**Problem focused coping** β=0.222 p<0.01	**Perceived severity** β=-0.406 p<0.01<br><br>**Attachment avoidance** β=-0.222 p<0.01<br><br>**Attachment anxiety β=**-0.302 p<0.01<br><br>**Pain catastrophizing** β=-0.686 p<0.01
Hansen et al., 2017 Denmark	Cross sectional<br><br>Evaluate the long-term effects of a mindfulness-based psychological intervention on chronic pain and QoL in endometriosis.	Exposure:<br>Mindfulness-based psychological intervention<br><br>Control: None<br><br>EHP-30 | SF-36<br>6-year follow-up	n=10<br><br>Mean age (SD) = 48 (10)	Mean (SD)<br><br>Pain: 52.53 (12.52)<br>Control and powerlessness: 65.28 (18.98)<br>Emotional Wellbeing: 52.08 (16.23)<br>Social support: 52.50 (25.89)<br>Self-image: 41.67 (21.52)<br>Work-life: 47.86 (29.94)<br>Relationship with children:	None	None

Author, year, country	Design / Purpose	Intervention or exposure potentially associated with QoL / QoL score and data point	Participants (n, age)	Level of quality of life at baseline	Factors significantly associated with improved quality of life	Factors significantly associated with a deterioration in quality of life
				46.43 (22.49)<br>Sexual intercourse: 66.67 (12.82)		
Khan et al., 2024 Trinidad and Tobago	Cross sectional<br><br>Assess how QoL is affected by the severity of pain and QoL among women with physician-diagnosed endometriosis in Trinidad and Tobago.	Exposure:<br>- Demographics<br>- Health<br>- Pain scale<br>- Acceptance of Illness<br><br>Control: None<br><br>WHOQOL-BREF<br>One data point	n=54<br>Mean age=38.65	Mean (SD)<br><br>Overall: 3.41 (0.84)<br><br>Psychological: 12.72 (2.72)<br>Physical : 12.29 (2.29)<br>Social : 11.88 (3.96)<br>Environment : 12.84 (3.46)	None	Mean difference p<br><br>**Poor health status**:<br>Psychological: 0.001<br>Physical : 0.009<br>Social : 0.018<br>Environment : 0.005
Kiykac et al., 2015 Turkey	Cross sectional<br><br>Investigate the effect of endometriotic symptoms, such as dysmenorrhea, dyspareunia and CPP on QoL in Turkish fertile patients.	Exposure:<br>- Dysmenorrhea (continuous VAS)<br>- Dyspareunia (continuous VAS)<br><br>Control: None<br><br>WHO-QOL-100<br>One data point	n=33<br><br>Mean age (SD) = 38.6 (5.3)	Mean (SD)<br><br>Physical: 71.3 (12.1)<br>Psychological: 72.4 (13.8)<br>Social: 71.7 (16.3)<br>Environmental: 69.0 (14)	None	**Dysmenorrhea (correlation):**<br>Physical domain: r = – 0.382, p = 0.028<br>Social domain: r = – 0.221, p = 0.013<br>Environmental domain: r = – 0.373, p = 0.033
Kold et al., 2012 Denmark	Prospective observational study<br><br>Assess the feasibility and effects of psychological intervention based on	Intervention: Psychological intervention based on mindfulness techniques, 10-session intervention (five individual and five group sessions, 15 hours in total	n=10<br><br>Mean age (SD) = NA	Mean (SD):<br><br>Pain: 52.53 (12.52)<br>Control and powerlessness: 65.28 (18.98)<br>Emotional wellbeing: 52.08 (16.23)<br>Social support: 52.50 (25.89)<br>Self image: 41.67 (21.52)	**Psychological intervention based on mindfulness techniques:**<br>Pain: dF= 3.4 F=18.06 p=0.000<br>Control and powerlessness: dF=3.24 F=15.64 p=0.000<br>Emotional wellbeing: dF=3.27 F=16.22 p=0.000<br>Social support: dF=3.27 F=4.89 p=0.008	None

Author, year, country	Design / Purpose	Intervention or exposure potentially associated with QoL / QoL score and data point	Participants (n, age)	Level of quality of life at baseline	Factors significantly associated with improved quality of life	Factors significantly associated with a deterioration in quality of life
	mindfulness techniques.	Control: None<br><br>EHP-30 | SF-36<br>Baseline | 6 months | 12 months				
Krabbenborg et al., 2021 Holland	Cross sectional<br><br>Obtain insight into the current diet of women with endometriosis, in terms of adherence to dietary guidelines, use of diets and perceived effects of dietary modifications.	Exposure: Dietary quality (DHD-15, 0-10, continuous)<br><br>Control: None<br><br>EHP-30<br>One data point	n=157<br><br>Mean age (SD) = 37.2 (7.1)	Mean (SD) = 193.6 (111.4) (out of 500)	None	None
Márki et al., 2017 Hungary	Cross sectional<br><br>Identify the main predictive factors of HRQoL and examine the effect of pain symptoms, emotion regulation and negative emotions on HRQoL in women with endometriosis.	Exposure:<br>- Anxiety (HADS - continuous)<br>- Depression (HADS - continuous)<br>- Stress (PSS - continuous)<br>- Emotional regulation (DERS - continuous)<br><br>Control: None<br><br>SF-36<br>One data point	n=193<br><br>Mean age (SD) = 33.87 (5.37)	No data	None	Correlation<br><br>**Anxiety**: r=-0.60 p<0.001<br>**Depression**: r=-0.54 p<0.001<br>**Stress**: r=-0.55 p<0.001<br>**Emotional regulation**: r=-0.38 p<0.001

Author, year, country	Design / Purpose	Intervention or exposure potentially associated with QoL / QoL score and data point	Participants (n, age)	Level of quality of life at baseline	Factors significantly associated with improved quality of life	Factors significantly associated with a deterioration in quality of life
Martins et al., 2021 Portugal	Cross sectional<br><br>Analyse the contribution of sociodemographic, clinical and psychological variables to QoL and sexual satisfaction in women with clinical endometriosis.	Exposure:<br>- Psychological morbidity (HADS - continuous)<br>- Sexual satisfaction (CSI-4, GMSEX - continuous)<br>- Marital satisfaction (continuous)<br>- Absence from work (continuous)<br>- Perception of symptom severity (continuous)<br>- CPP intensity (continuous)<br>- Dyspareunia intensity (continuous)<br>- Dyschezia intensity (continuous)<br><br>Control: None<br><br>EHP-30<br>One data point	n=124<br><br>Mean age (SD) = 36.18 (6.51)	No data	**Higher sexual satisfaction**: β=-0.221 p<0.05<br><br>**Marital satisfaction**: β=0.193 p<0.05	**More absence from work**: β=0.245 p<0.01<br><br>**Higher perception of symptom severity**: β=0.298 p<0.01<br><br>**Higher dyschezia**: β=0.280 p<0.01<br><br>**Higher dyspareunia**: β=0.248 p<0.01<br><br>**Higher CPP**: β=0.306 p<0.01<br><br>**More psychological morbidity**: β=0.658 p<0.001
Matasariu et al., 2017 Romania	Cross sectional<br><br>Explore some of the psychological reactions associated with endometriosis and depression.	Exposure:<br>- Infertility (FPI 0-100 - continuous)<br>- Depression (BDI - continuous)<br><br>Control: None<br><br>EHP-30<br>One data point	n=205<br><br>Mean age (SD) = 35.46 (2.34)	No data	None	**Qualitatively reported stress**
Matías-González	Cross sectional	Exposure:<br>- Stigma (CogApp, SSE)	n=169	No data	**Social support**: β=-0.206, p=0.031	**Incapacitating pain**: β=0.266, p<0.001

Author, year, country	Design / Purpose	Intervention or exposure potentially associated with QoL / QoL score and data point	Participants (n, age)	Level of quality of life at baseline	Factors significantly associated with improved quality of life	Factors significantly associated with a deterioration in quality of life
et al., 2022 Puerto Rico	Understand the role of endo-stigma on the QoL among a sample of people living with endometriosis in Latin America and the Caribbean.	- Social support<br>- Pain disrupt day-to-day functioning<br><br>Control: None<br><br>EHP-5<br>One data point	Mean age (SD) = 33.15 (7.44)			**Endometriosis stigma**: β=0.339, p=<0.001
Meissner et al., 2016 Germany	RCT<br><br>Evaluate whether psychotherapy with somatosensory stimulation is effective for the treatment of pain and QoL in patients with endometriosis related pain.	Intervention: Psychological intervention - Psychotherapy with somatosensory stimulation - 30–60 minutes<br><br>Control: waiting list<br><br>SF-12<br>Baseline | 3 months | 6 months	Intervention:<br>n=35<br>Mean age (95CI) = 35.0 (33.3–36.6)<br><br>Control:<br>n=32<br>Mean age (95CI) = 36.2 (34.5–37.9)	Mean (95CI)<br><br>Intervention:<br>Physical = 46.5 (43.9–49.0)<br>Mental 40.6 (36.4–44.9)<br>Functional 18.5 (16.1–20.9)<br><br>Control:<br>Physical = 42.2 (39.6–44.8)<br>Mental = 40.9 (37.4–44.4)<br>Functional = 19.0 (16.1–21.8)	**Psychotherapy with somatosensory stimulation (3-month, MD):**<br>Physical: 3.8 (CI 0.5–7.1) p=0.026<br>Mental: 5.9 (CI 0.6–11.3) p=0.031	None
Merlot et al., 2023 France	RCT<br><br>Assess the effects of repeated at-home administrations of a 20-minute virtual reality (VR) solution (Endocare) compared with	Intervention: Virtual reality intervention - 20-minute treatment<br><br>Control: Sham<br><br>EHP-5<br>Baseline | Daily for 6 days	Intervention:<br>n=51<br><br>Control:<br>n=51<br><br>Mean age (SD) = 32.9 (6.96)	Mean (SD)<br><br>Intervention: 44.8 (23.7)<br><br>Control: 42.8 (21.8)	None	None

Author, year, country	Design / Purpose	Intervention or exposure potentially associated with QoL / QoL score and data point	Participants (n, age)	Level of quality of life at baseline	Factors significantly associated with improved quality of life	Factors significantly associated with a deterioration in quality of life
	a sham condition on pain in women experiencing pelvic pain due to endometriosis.					
Miazga et al., 2024 Canada	Interventional<br><br>Assess the effectiveness of a virtual mindfulness-based stress reduction (MBSR) program to improve quality of life and pain in people with endometriosis.	Intervention: Virtual mindfulness-based stress reduction program: 8 weeks, 1 session/week, 2.5h/session<br><br>Control: None<br><br>EHP-30<br>Baseline | 8 weeks	n=10<br>Mean age (SD)=32.53 (5.87)	Mean (SD)<br><br>Pain: 49.09 (25.38)<br>Control and Powerlessness: 70.42 (21.20)<br>Emotional Well-Being: 52.92 (24.22)<br>Social Support: 66.25 (22.67)<br>Self-Image: 85.00 (23.17)	**Virtual mindfulness-based stress reduction program**<br>(Mean difference p)<br>control and powerlessness: p=0.012<br>emotional well-being: p=0.048<br>social support p=0.030<br>self-image p=0.014	None
Mira et al., 2020 Brazil	RCT<br><br>Evaluate the clinical effectiveness of complementary treatment using self-applied electrotherapy treatment for pain control over the standard hormonal treatment alone for deep	Intervention: Electrotherapy (TENS), twice a day, 20 min per application, for 8 weeks<br><br>Control: Hormonal therapy only<br><br>EHP-30<br>Baseline | 8 weeks	Intervention:<br>n=53<br>Mean age (SD) = 35.06 (6.17)<br><br>Control:<br>n=48<br>Mean age (SD) = 37.21 (6.51)	Mean (SD)<br><br>Intervention: 43.97 (17.03)<br><br>Control: 37.06 (16.03)	None	None

Author, year, country	Design / Purpose	Intervention or exposure potentially associated with QoL / QoL score and data point	Participants (n, age)	Level of quality of life at baseline	Factors significantly associated with improved quality of life	Factors significantly associated with a deterioration in quality of life
	infiltrative endometriosis (DIE).					
Montanari et al., 2013 Italy	Cross sectional<br><br>Evaluate sexual function in women with deep infiltrating endometriosis (DIE) and to study the impact of endometriosis symptoms and type of lesion on patients' sexual function.	Exposure: Sexual function (SHOW-Q)<br><br>Control: None<br><br>SF-36<br>One data point	n=182<br><br>Mean age (SD) = 34.40 (5.42)	No data	**Better sexual function:**<br>Correlation r=0.32, 95%CI=[0.18, 0.45], P < 0.0001	None
Muselli et al., 2024 Italy	Cross sectional<br><br>Investigate the relationship between unhealthy behaviors (physical inactivity, tobacco consumption, and alcohol consumption) and health-related quality of life.	Exposure:<br>- unhealthy behaviors (physical inactivity, tobacco consumption, and alcohol consumption) (dichotomous)<br>- age groups (3 categories)<br>- marital status (2 categories)<br>- educational level (2 categories)<br>- employment (dichotomous)<br>- living alone (dichotomous)<br>- comorbidities (dichotomous)	n=1045<br>Age n (%)<br>18-34yo: 491 (47.0%)<br>35-44 yo: 435 (41.6%)<br>>=45yo: 119 (11.4%)	Mean (SD)<br><br>Physical wellbeing: 66.51 (27.24)<br>Physical role limitations: 40.26 (41.14)<br>Bodily Pain: 46.24 (27.63)<br>General: 37.09 (20.73)<br>Fatigue/Vitality: 35.54 (18.45)<br>Social: 44.28 (25.53)<br>Emotional role limitations: 39.26 (38.92)<br>Emotional well-being: 47.57 (19.19)	Mean difference p<br><br>**Employment (vs. not employed)**<br>Physical wellbeing: p<0.01<br>Physical role limitations: p<0.01<br>Bodily Pain: p<0.01<br>General: p<0.01<br>Fatigue/Vitality: p<0.01<br>Social: p<0.01<br>Emotional role limitations: p<0.01<br>Emotional well-being: p<0.01	Mean difference p<br><br>**Tobacco smoking (vs. no smoking):**<br>Physical wellbeing: p<0.001<br>Physical role limitations: p=0.001<br>Bodily Pain: p=0.002<br>General: p=0.003<br>Fatigue/Vitality: p=0.012<br>Social: p=0.005<br>Emotional role limitations: p=0.001<br>Emotional well-being: p=0.004<br><br>**Physical inactivity (vs. physical activity)**<br>Physical wellbeing: p<0.001<br>Physical role limitations:

Author, year, country	Design / Purpose	Intervention or exposure potentially associated with QoL / QoL score and data point	Participants (n, age)	Level of quality of life at baseline	Factors significantly associated with improved quality of life	Factors significantly associated with a deterioration in quality of life
		- disease duration (3 categories)<br><br>Control: None<br><br>SF-36<br>One data point				p=0.003<br>Bodily Pain: p<0.001<br>General: p<0.001<br>Fatigue/Vitality: p<0.001<br>Social: p<0.001<br>Emotional role limitations: p<0.001<br>Emotional well-being: p<0.001<br><br>**Comorbidities (vs. no)**<br>Physical wellbeing: p<0.001<br>Physical role limitations: p=0.032<br>Bodily Pain: p<0.001<br>General: p<0.001<br>Fatigue/Vitality: p<0.001<br>Social: p<0.001<br>Emotional role limitations: p<0.001<br>Emotional well-being: p=0.010
Norman et al., 2021 USA	Mixed method<br><br>Identify self-care interventions women living in the United States are using to manage endometriosis-related pain, describe frequency of use, and determine perceived effectiveness.	Exposure:<br>- Confidence managing symptoms (continuous 0-10)<br>- Number of self-care strategies used more than once a week (continuous)<br>- Typical endometriosis-related pain intensity (continuous 0-10)<br><br>Control: None<br><br>EHP-30<br>One data point	n=98<br><br>Mean age (SD) = 33.39 (6.43)	Mean (SD)<br><br>Pain: 55.26 (22.95)<br>Control and Powerlessness: 70.03 (27.16)<br>Emotional Well-Being: 66.24 (25.87)<br>Social Support: 61.42 (26.30)<br>Self-Image: 66.24 (25.87)	Correlation<br><br>**Increase confidence managing symptoms:**<br>Pain: r=-0.48, p<0.01<br>Control and Powerlessness: r=-0.55, p<0.01 Emotional Well-Being: r=-0.37, p<0.01<br>Social Support: r=-0.47, p<0.01<br>Self image: r=-0.50, p<0.01	Correlation<br><br>**Pain intensity:**<br>Pain: r=0.49, p<0.01<br>Control and Powerlessness: r=0.38, p<0.01 Emotional Well-Being: r=0.28, p<0.05<br>Social Support: r=0.31, p<0.01<br><br>**Increase number of self-care strategies used more than once per week:**<br>Pain: r=0.44, p<0.01<br>Control and Powerlessness: r=0.34, p<0.01 Emotional Well-Being: r=0.32, p<0.01<br>Social Support: r=0.32, p<0.01

Author, year, country	Design / Purpose	Intervention or exposure potentially associated with QoL / QoL score and data point	Participants (n, age)	Level of quality of life at baseline	Factors significantly associated with improved quality of life	Factors significantly associated with a deterioration in quality of life
						Self image: r=0.31, p<0.01
Pessoa de Farias Rodrigues et al., 2020 Brazil	Cross sectional<br><br>Verify the levels of QoL in women with endometriosis and infertility; and to compare these levels between staging groups as well as the clinical symptoms of endometriosis with aggravating factors of infertility.	Exposure:<br>- Stage of endometriosis (I/II vs III/IV)<br>- Dyspareunia (localization)<br>- Degree of pain (5 categories)<br>- CPP (dichotomous)<br><br>Control: None<br><br>SF-36<br>One data point	Stage I/II:<br>n=26<br>Mean age (SD) = 35.27 (3.64)<br><br>Stage III/IV:<br>n=80<br>Mean age (SD) = 34.04 (3.39)	Mean (SD)<br><br>Stage I/II:<br>Vitality: 54.42 (14.72)<br>Mental health: 59.54 (21.18)<br>General health: 58.69 (16.56)<br>Social functioning: 66.34 (26.40)<br><br>Stage III/IV:<br>Vitality: 56.24 (11.38)<br>Mental health: 59.23 (18.52)<br>General health: 60.54 (17.57)<br>Social functioning: 66.20 (23.58)	None	Results for women with endometriosis AND infertility<br><br>**Dyspareunia**<br>Physical functioning: p = 0.017<br>Role-emotional: p = 0.013<br>General health: p = 0.001<br><br>**Pain**<br>Physical functioning: p = 0.005<br>Role-physical: p = 0.011<br><br>**CPP**<br>Bodily pain: p = 0.017
Pinot-Monange et al., 2019 France	Prospective study<br><br>Assess the feasibility and effect of repetitive transcranial magnetic stimulation therapy (rTMS) to reduce pain and improve QoL in patients with CPP due to endometriosis.	Intervention:<br>Repetitive transcranial magnetic stimulation therapy (rTMS), one session per day for 5 consecutive days during one month, each session consisted of 1.500 pulses at 10 Hz.<br><br>Control: None<br><br>EHP-30 | SF-36<br>Baseline (28 days before treatment) | 8 days after treatment |	n=12<br><br>Mean age (SD) = 38 (8)	Mean (SD)<br><br>SF36:<br>Physical: 37.5 (7.9)<br>Mental: 36.8 (10.6)<br><br>EHP-30:<br>Emotional well-being: 54.9 (15.5)<br>Pain levels: 65.0 (16.3)	Mean (SD)<br><br>**Repetitive Transcranial Magnetic Stimulation Therapy** (at 8 days):<br>Physical: 37.5 (7.9) vs. 42.0 (9.7); p = 0.047<br>Emotional well-being: 54.9 (15.5) vs. 43.1 (19.6); p = 0.02	None

Author, year, country	Design / Purpose	Intervention or exposure potentially associated with QoL / QoL score and data point	Participants (n, age)	Level of quality of life at baseline	Factors significantly associated with improved quality of life	Factors significantly associated with a deterioration in quality of life
		28 days post treatment				
Pontoppidan et al., 2023 Sweden	Cross sectional<br><br>Investigate how women with endometriosis perceive their QoL, and to analyze which clinical factors are associated with QoL.	Exposure:<br>- age at first symptoms of endometriosis (continuous)<br>- diagnostic delay (continuous)<br>- endometriosis severity (continuous)<br>- >10 visits to general practitioners before referral to a gynecologist (dichotomous)<br>- having a responsible gynecologist to care for endometriosis (dichotomous)<br>- ever tried to conceive for > 12 months (dichotomous)<br>- previous or current mental health issues (dichotomous)<br>- patient-centeredness (continuous)<br><br>Control: None<br><br>EHP-30<br>One data point	n=476<br>Mean age (SD)=36.5 (9.0)	Mean (SD)=45.9 (24.5)	B p<br><br>**Later age at first onset of symptoms**: B=-0.64 p<0.001<br><br>**Patient-centeredness**: B=-2.59 p<0.001	B p<br><br>**Having more than 10 visits to general practitioners before referral to a gynecologist**: B=5.58 p=0.038<br><br>**Current or previous mental health issues**: B=7.98 p<0.01
Ravins et al., 2023	AB design study	Intervention: Yoga for endometriosis, online	n=42	Mean (SD): 57.87 (17.2)	**Endometriosis yoga:**<br>MD (SD) = 10.43 (2.59) p	None

Author, year, country	Design / Purpose	Intervention or exposure potentially associated with QoL / QoL score and data point	Participants (n, age)	Level of quality of life at baseline	Factors significantly associated with improved quality of life	Factors significantly associated with a deterioration in quality of life
Israel	Examine the effect of practicing endometriosis yoga on the stress and QoL of women diagnosed with endometriosis.	intervention (8 weeks of two 90-minute sessions a week)<br><br>Control: None<br><br>EHP-30<br>Baseline (two months before treatment) | 2 months after treatment | 4 months	Mean age (SD) = 30.42 (7.7)		=0.001	
Rees et al., 2022 UK	Cross sectional<br><br>Investigate the role of pain self-efficacy, HLOC [health locus of control], coping style and illness uncertainty on women's QoL.	Exposure:<br>- Self efficacy (PSEQ, continuous)<br>- Health locus of control (HLOC) / perceived degree of control (continuous)<br>- Coping style (continuous)<br>- Illness uncertainty (continuous)<br><br>Control: None<br><br>WHOQOL-BREF<br>One data point	n=230<br><br>Mean age = 31	Mean (SD)<br><br>Physical health: 46.30 (19.30)<br>Psychological health: 48.00 (15.10)<br>Social relationships: 49.00 (23.80)<br>Environment: 60.02 (16.67)	**Pain self-efficacy:**<br>Physical: β=1.108 p<0.001<br>Psychological: β=0.548 p<0.001<br>Social: β=0.530 p<0.002<br>Environment: β=0.518 p<0.005	**Illness uncertainty:**<br>Physical: β=0.211 p<0.019<br>Psychological: β=0.182 p<0.035
Rohloff, Götz et al., 2024 Germany	RCT<br><br>Examine the impact of the Endo-App (Endo Health GmbH, Chemnitz, Germany) on both disease-related quality	Intervention: 12 weeks of active use of the Endo-App as an add-on to the ongoing treatment of standard care<br><br>Control: Standard care<br><br>EHP-30 and EHP-5	n=122<br>Mean age=29.6<br><br>Intervention: n=34<br><br>Control: n=64	Not available	**Endo-App**<br><br>Mean difference p between T0 and T12 vs. control<br><br>EHP-5: -16.76 p=0.008<br><br>EHP-30: -15.48 p=0.004	None

Author, year, country	Design / Purpose	Intervention or exposure potentially associated with QoL / QoL score and data point	Participants (n, age)	Level of quality of life at baseline	Factors significantly associated with improved quality of life	Factors significantly associated with a deterioration in quality of life
	of life and symptoms of endometriosis affecting it.	Baseline | 4 weeks | 8 weeks | 12 weeks				
Rohloff, Rothenhöfer et al., 2024 Germany	Interventional<br><br>Examine whether there is evidence of beneficial effects of the smartphone app "Endo-App®" and whether a multicenter randomized controlled trial should be planned to substantiate these effects.	Intervention: 2 weeks of use of Endo-App, digital health application specifically designed to support the multimodal therapy of endometriosis<br><br>Control: Non user group<br><br>EHP-30<br>Baseline | 2 weeks	n=106<br>Mean age (range)=33 (20-51)<br><br>Intervention:<br>n=64<br>Mean age (range)=33 (20-51)<br><br>Control:<br>n=42<br>Mean age (range)=32 (22-46)	Mean (SD)<br><br>Intervention: 58.43 (18.44)<br><br>Control: 44.54 (19.56)	**Endo-App**<br>Mean difference p:<br><br>Intervention: -9.06 p=0.0000<br><br>Control: -1.98 p=0.3200	None
Sepulcri et al., 2009 Brazil	Prospective study<br><br>Assess depressive symptoms, anxiety and QoL in women with pelvic endometriosis.	Exposure:<br>- Age (continuous)<br>- Duration of typical endometriosis complaint (continuous)<br>- Intensity of pain (continuous)<br>- Mean time of diagnosis (continuous)<br>- Stages<br>- Infertility (dichotomous)<br><br>Control: None	n=104<br><br>Mean age (SD) = 34.6 (6.3)	Median<br><br>Physical health: 48.2<br>Psychological health: 50<br>Social relationships: 50<br>Environment: 54.7	None	**Intensity of pain:**<br>Physical: r=-0.2344 p=0.017<br><br>**Duration of treatment:**<br>Physical: r=-0.2337 p=0.017

Author, year, country	Design / Purpose	Intervention or exposure potentially associated with QoL / QoL score and data point	Participants (n, age)	Level of quality of life at baseline	Factors significantly associated with improved quality of life	Factors significantly associated with a deterioration in quality of life
		WHOQOL-BREF<br>One data point				
Sesti et al., 2007 Italy	RCT<br><br>Evaluate the effectiveness for the outcomes of endometriosis-related pain and QoL of conservative surgery plus placebo compared with conservative surgery plus hormonal suppression treatment or dietary therapy.	Intervention:<br>- GNRH-a for 6 months (triptorelin or leuprorelin, 3.75 mg every 28 days)<br>- Continuous estroprogestin for 6 months (ethynilestradiol, 0.03 mg plus gestoden, 0.75 mg)<br>- Dietary therapy for 6 months<br><br>Control: Placebo<br><br>SF-36<br>Baseline (seven days before conservative surgery) | 12 months	GNRH-a:<br>n=39<br>Mean age (SD) = 30.0 (3.7)<br><br>Continuous estroprogestin:<br>n=38<br>Mean age (SD) = 30.6 (3.6)<br><br>Dietary therapy:<br>n=35<br>Mean age (SD) = 29.0 (3.9)<br><br>Control:<br>n=110<br>Mean age (SD) = 31.0 (4.0)	No precise data	(no statistical data available)<br><br>**GNRH analogue**<br><br>**Continuous monophasic oral contraceptive**<br><br>**Dietary therapy**	None
Simonsen et al., 2019 Denmark	Mixed method<br><br>Assess if the implementation of the Guided Self Determination method targeted women with complex endometriosis appeared feasible and supported self-management.	Intervention: Guided Self-Determination method (five conversations based on pre-filled disease-specific reflection sheets, semi-structured, telephone interviews and focus group interviews)<br><br>Control: None<br><br>EHP-30<br>Baseline | 2 weeks | 1	n=10 (at baseline and 2 weeks)<br>n=4 (at 1 year)<br><br>Age:<br>20-25 yo = 1/10<br>25-30 yo = 4/10<br>30-35 yo = 1/10<br>35-40 yo = 2/10<br>40-45 to = 2/10	Median:<br><br>Pain: 55<br>Control and powerlessness: 73<br>Emotional well-being: 60<br>Social support: 78<br>Self-image: 67	Median difference<br><br>**Guided Self-Determination method** (at 2 weeks):<br>Pain = -14<br>Control and powerlessness =-19<br>Emotional well-being = -17<br>Social support = -22<br>Self-image = -25	None

Author, year, country	Design / Purpose	Intervention or exposure potentially associated with QoL / QoL score and data point	Participants (n, age)	Level of quality of life at baseline	Factors significantly associated with improved quality of life	Factors significantly associated with a deterioration in quality of life
		year				
Škegro et al., 2021 Croatia	Cross sectional<br><br>Assess HRQoL and mental health status in patients with endometriosis, investigating also their relationship with endometriosis-related comorbid symptoms and conditions, such as pain and infertility.	Exposure:<br>- Education (2 categories)<br>- Marriage (3 categories)<br>- Mental health (continuous)<br>- Pain (continuous)<br>- Infertility (dichotomous)<br><br>Control: None<br><br>EHP-5<br>One data point	n=79<br><br>Mean age (SD) = 35.03 (7.11)	Mean (SD) = 37.03 (22.08)	None	Correlation<br><br>**Depression**: r=0.515<br>**Stress**: r=0.558<br>**Pain**: r=0.565<br>**Anxiety**: r=0.295<br>**Infertility**: r=0.267
Skinner et al., 2024 New Zealand	Cross sectional<br><br>Examine the relationship between self-compassion and HRQoL in individuals with endometriosis in Aotearoa New Zealand.	Exposure: Self-compassion (continuous)<br><br>Control: None<br><br>EHP-30<br>One data point	n=603<br>Mean age (SD)=29.48 (6.84)	Mean (SD)=56.49 (20.21)	**Higher level of self-compassion**<br>B (CI)= -8.56 (-11.04;-6.08)	None
Soliman et al., 2017 USA	Cross sectional<br><br>Examine the symptomatic burden of endometriosis on HRQoL in	Exposure:<br>- Number of symptoms (continuous)<br>- Age (5 ranges)<br><br>Control: None	n=1,269<br><br>Mean age (SD) = 34.3 (0.3)	Mean (SD)<br><br>Pain: 34.8 (1.1)<br>Control and powerlessness: 37.8 (1.2)<br>Emotional well-being: 34.8 (1.1)	None	MD<br><br>**Increase in the number of symptoms**: $p < 0.0001$ for all subscales<br><br>**Young age**: 18-29 years old vs.

Author, year, country	Design / Purpose	Intervention or exposure potentially associated with QoL / QoL score and data point	Participants (n, age)	Level of quality of life at baseline	Factors significantly associated with improved quality of life	Factors significantly associated with a deterioration in quality of life
	women in the United States (US).	EHP-30<br>One data point		Social support: 33.6 (1.1)<br>Self-image: 34.3 (1.2)		40-44, p<0.01 for all subscales
Spinoni et al., 2024 Italy	Cross sectional<br><br>Evaluate whether the impact of endometriosis-related chronic pelvic pain on women's health-related quality of life is influenced by cognitive factors (i.e., illness representations and coping strategies).	Exposure:<br>- Negative illness representations (continuous)<br>- Maladaptive coping strategies (continuous): catastrophizing and illness-focused coping<br>- Chronic pelvic pain (continuous)<br>- Age (continuous)<br>- Diagnostic delay (continuous)<br>- BMI (continuous)<br><br>Control: None<br><br>EHP-30<br>One data point	n=273<br>Mean age (SD)=35.3 (7.31)	Not available	None	Correlation p<br><br>**Diagnostic delay**: 0.18 p<0.05<br><br>**BMI**: 0.51 p<0.01<br><br>**Negative illness representations**: 0.56 p<0.01<br><br>**Maladaptive coping strategies (illness-focused coping, catastrophizing)**: 0.87 p<0.01 and 0.52 p<0.01<br><br>**Chronic pelvic pain**: 0.58 p<0.01
Stochino Loi et al., 2019 Italy	Interventional<br><br>Evaluate the effectiveness of the ultramicronized-palmitoylethanolamide (um-PEA) and co-micronised palmitoylethanolamide/polydatin m(PEA/PLD) in the	Intervention: Supplement ultramicronized-palmitoylethanolamide and co-micronized palmitoylethanolamide/polydatin - um-PEA twice daily for 10 days followed by m(PEA/PLD) twice daily for 80 days<br><br>Control: None	n=30<br><br>Mean age (SD) = NA	Mean (SD)<br><br>Physical functioning = 67 (26)<br>Body pain = 54 (21)<br>Role limitations due to physical health problems = 36 (26)<br>Role limitations due to personal or emotional problems = 45 (36)<br>Emotional well-being = 53 (17)<br>Social functioning = 61 (26)	MD<br><br>**Supplement ultramicronized-palmitoylethanolamide and co-micronized palmitoylethanolamide/polydatin**: p<0.0005 for all subscales	None

Author, year, country	Design / Purpose	Intervention or exposure potentially associated with QoL / QoL score and data point	Participants (n, age)	Level of quality of life at baseline	Factors significantly associated with improved quality of life	Factors significantly associated with a deterioration in quality of life
	management of CPP related to endometriosis in patients desiring pregnancy.	SF-36<br>Baseline | 90 days after treatments		Energy/fatigue = 46 (18)<br>General health perceptions = 59 (20)		
Sullivan-Myers et al., 2021 Australia	Cross sectional<br><br>Analyze the relationship between specific QoL domains and depression, anxiety, and stress in the endometriosis population.	Exposure:<br>- Depression (categories)<br>- Anxiety (categories)<br>- Stress (categories)<br><br>Control: None<br><br>EHP-30 | SF-36<br>One data point	n=584<br><br>Mean age (SD) = 31.2 (7.50)	Mean (SD)<br><br>EHP-30:<br>Pain: 55.35 (20.30)<br>Control & Powerlessness: 71.09 (23.21)<br>Social support: 65.33 (22.85)<br>Self image: 67.35 (24.78)<br><br>SF-36:<br>Physical functioning: 65.63 (25.84)<br>Role limitations due to physical health: 22.56 (33.83)<br>Vitality: 24.36 (17.08)<br>Social Functioning: 45.86 (24.84)	None	Correlation<br><br>**Depression**: p<0.01 for all subscales<br><br>**Anxiety**: p<0.01 for all subscales<br><br>**Stress**: p<0.01 for all subscales
Thabet et al., 2018 Saudi Arabia	RCT<br><br>Assess the effectiveness of pulsed high-intensity laser therapy on pain, adhesions, and QoL in women with endometriosis.	Intervention: Pulsed High-Intensity Laser Therapy three times per week for 8 weeks<br><br>Control: Sham intervention<br><br>EHP-5<br>Baseline | 8 weeks	Intervention: n=20<br><br>Control: n=20<br><br>Age range: 24-32 yo	No data	**Pulsed High-Intensity Laser Therapy**: MD = 0.50 after treatment, p=0.0001	None
Thammasiri et al.,	Cross sectional	Exposure:<br>- Pain score:	n=99	Mean (SD) = 35.0 (15.9)	None	**CPP**: β=7.53 CI=1.85;13.20

Author, year, country	Design / Purpose	Intervention or exposure potentially associated with QoL / QoL score and data point	Participants (n, age)	Level of quality of life at baseline	Factors significantly associated with improved quality of life	Factors significantly associated with a deterioration in quality of life
2022<br>Thailand	Evaluate the QoL of women with endometrioma and explore the associated factors.	dyspareunia, CPP (continuous and 3 categories)<br>- Age (continuous)<br>- Married (3 categories)<br>- BMI (continuous)<br>- Age of first diagnosis (continuous)<br>- Time since of first symptom to diagnosis (continuous)<br><br>Control: None<br><br>EHP-30<br>One data point (before treatment for ovarian endometrioma)	Mean age (SD) = 35.2 (7.4)			**Pain score**: β=2.79 CI=1.48;4.18
Touboul et al., 2013 France	Cross sectional<br><br>Evaluate the QOL of patients with deep infiltrating endometriosis (DIE) using EuroQOL (EQ-5D) and its correlation with symptoms and locations of endometriotic lesions.	Exposure:<br>- Dysmenorrhea (0-10 - continuous)<br>- Dyspareunia (0-10 - continuous)<br>- Cyclic Pelvic pain (0-10 - continuous)<br>- Painful defecation (0-10 - continuous)<br>- Gastrointestinal disorders (dichotomous)<br><br>Control: None<br><br>EQ-5D-3L | EQ-5D VAS<br>One data point	n=159<br><br>Mean age (SD) = 34.8 (6.6)	Mean (SD)<br><br>EQ-5D: 77 (14)<br><br>Health state: 63.4 (21)	None	MD (EQ-5D-3L | EQ-5D VAS)<br><br>**Dysmenorrhea**: p=0.01 | p=0.03<br><br>**Dyspareunia**: p<0.001 | p<0.01<br><br>**Cyclic Pelvic pain**: p<0.001 | p<0.001<br><br>**Painful defecation**: p<0.001 | p=0.01<br><br>**Gastrointestinal disorders**: p<0.001 | p<0.01

Author, year, country	Design / Purpose	Intervention or exposure potentially associated with QoL / QoL score and data point	Participants (n, age)	Level of quality of life at baseline	Factors significantly associated with improved quality of life	Factors significantly associated with a deterioration in quality of life
		(before any potential surgical treatment of DIE)				
Van Haaps et al., 2023 The Netherlands	Interventional<br><br>Determine the influence of dietary interventions, namely the low fermentable oligo-, di-, mono-saccharides, and polyols (Low FODMAP) diet and endometriosis diet, on endometriosis-related pain and quality of life (QoL) compared to a control group.	Intervention:<br>- Low FODMAP diet for 6 months<br>- Endometriosis diet for 6 months<br><br>Control: No diet for 6 months<br><br>EHP-30<br>Baseline | 3 months | 6 months	n=62<br><br>Intervention:<br>- Low FODMAP diet:<br>n=22<br>Mean age (SD)=36.9 (5.9)<br>- Endometriosis diet:<br>n=21<br>Mean age (SD)=39.1 (15.8)<br><br>Control:<br>n=19<br>Mean age (SD)=37.6 (8.5)	Mean (SD)<br><br>Intervention (Low FODMAP or endometriosis diet):<br>Pain: 42.0 (29.0)<br>Control and Powerlessness: 50.0 (30.2)<br>Emotional Well-Being: 33.3 (33.3)<br>Social Support: 34.4 (39.1)<br>Self-Image: 50.0 (43.8)<br><br>Control:<br>Pain: 28.4 (35.5)<br>Control and Powerlessness: 35.4 (31.3)<br>Emotional Well-Being: 31.3 (26.0)<br>Social Support: 43.8 (39.1)<br>Self-Image: 41.7 (27.1)	**Low FODMAP or endometriosis diet**:<br>Mean difference (CI) p vs. control<br>Social support: -11.26 (-18.77;-3.74) p=0.004	None
Van Niekerk et al., 2022 Australia	Cross sectional<br><br>Examine the relationships between endometriosis-related symptom experience and HRQoL.	Exposure:<br>- Age (continuous)<br>- Symptom duration, burden, and distress (continuous)<br>- Depression (continuous)<br>- Pain, dysmenorrhea, clitoral pain, dyspareunia (continuous)<br>- Bloating	n=318<br><br>Mean age (SD) = 30.75 (7.55)	Mean (SD)<br><br>Control and powerlessness: 65.68 (25.80)<br>Self-image: 61.29 (29.27)<br>Social support: 60.30 (26.21)<br>Pain: 54.99 (23.53)<br>Emotional wellbeing: 49.75 (23.47)	**Age**:<br>Pain: β=-0.16 p=0.033 | Self-image: β=-0.26 p=0.001	**Level of current endometriosis-related pain**<br>Pain: β=0.25 p=0.001 | Control/powerlessness: β=0.19 p=0.001 | Social support: β=0.14 p=0.012 | Self-image: β=0.12 p=0.028<br><br>**Level of somatic concern**<br>Pain: β=0.30 p=0.001 | Control/powerlessness: β=0.21 p=0.001 | Emotional

Author, year, country	Design / Purpose	Intervention or exposure potentially associated with QoL / QoL score and data point	Participants (n, age)	Level of quality of life at baseline	Factors significantly associated with improved quality of life	Factors significantly associated with a deterioration in quality of life
		(continuous)<br><br>Control: None<br><br>EHP-30<br>One data point				wellbeing: β=0.21 p=0.001 | Self-image: β=0.33 p=0.000<br><br>**Level of depression**<br>Control/powerlessness: β=0.28 p=0.001 | Emotional wellbeing: β=0.45 p=0.000 | Social support: β=0.39 p=0.000 | Self-image: β=0.16 p=0.019<br><br>**Dysmenorrhea**<br>Pain: β=0.19 p=0.001 | Control/powerlessness: β=0.15 p=0.006<br><br>**Diagnostic delay**<br>Control/powerlessness: 0.11 p=0.019<br><br>**Duration of endometriosis-related symptoms**<br>Self-image: β=0.21 p=0.008<br><br>**Clitoral pain**<br>Emotional wellbeing: β=0.13 p=0.003<br><br>**Dyspareunia**<br>Social support: β=0.19 p=0.001<br><br>**Bloating**<br>Self-image: β=0.22 p=0.000
Van Niekerk et al., 2023 Australia	Cross sectional<br><br>Examine the influence of self and body	Exposure: Self and body compassion (3 categories)<br><br>Control: None	n=318<br><br>Mean age (SD) = 30.8 (7.6)	Mean (SD)<br><br>Physical wellbeing: 44.83 (19.76)<br>Emotional well-being: 44.14	**Self compassion**<br>Emotional: β=0.41 p=0.002<br><br>**Body compassion**<br>Physical: β=0.41 p=0.001	None

Author, year, country	Design / Purpose	Intervention or exposure potentially associated with QoL / QoL score and data point	Participants (n, age)	Level of quality of life at baseline	Factors significantly associated with improved quality of life	Factors significantly associated with a deterioration in quality of life
	compassion on HRQoL in people diagnosed with endometriosis.	SF-36<br>One data point		(21.71)<br>Physical: 69.31 (23.77)<br>Physical role limitations: 23.74 (35.28)<br>Emotional: 51.65 (17.25)<br>Emotional role limitations: 31.55 (37.35)<br>Fatigue/Vitality: 25.46 (16.01)<br>Social: 49.21 (24.61)<br>Bodily Pain: 43.17 (22.75)<br>General: 38.73 (21.02)	Emotional: β=0.20 p=0.012<br>General: β=0.32 p=0.001<br>Bodily pain: β=0.28 p=0.001<br>Fatigue: β=0.31 p=0.001<br>Social: β=0.41 p=0.001	
Wu et al., 2023 Taiwan	Cross sectional<br><br>Assess the health-related quality of life and identify its associated factors in women with endometriosis.	Exposure:<br>- Age (continuous)<br>- Marital status (dichotomous)<br>- Educational attainment (3 categories)<br>- Family income (3 categories)<br>- Age at menarche (continuous)<br>- Menstrual cycle (3 categories)<br>- Period length (3 categories)<br>- Perceived menstrual flow (3 categories)<br>- Symptom distress (continuous)<br>- Self-management strategies (continuous)<br><br>Control: None	n=216<br>Mean age (SD)=35.62 (6.02)	Mean<br><br>Pain: 46.43<br>Control and Powerlessness: 56.14<br>Emotional Well-Being: 48.3<br>Social Support: 49.52<br>Self-Image: 43.70	None	Correlation p<br><br>**Lower educational attainment**: 6.61 p<0.01<br>**Earlier age at menarche**: 0.39 p<0.001<br>**Shorter menstrual cycle**: 4.81 p<0.01<br>**Longer period length**: 25.31 p<0.001<br>**Perceived intensity menstrual flow**: 9.53 p<0.001<br>**Symptom distress**: 0.78 p<0.001<br>**Frequent self-management strategies** 0.51 p<0.001

Author, year, country	Design / Purpose	Intervention or exposure potentially associated with QoL / QoL score and data point	Participants (n, age)	Level of quality of life at baseline	Factors significantly associated with improved quality of life	Factors significantly associated with a deterioration in quality of life
		EHP-5<br>One data point				
Yazdankhah et al., 2025 Iran	Interventional<br><br>Assess the effects of a lemongrass oil vaginal cream on pain levels, mass size, and overall health profiles in endometriosis patients.	Intervention: Vaginal Cream from Cymbopogon citratus Oil, 5 g of the cream with a vaginal applicator on nights without menstrual bleeding: 21 applications / month during 2 months<br><br>Control: None<br><br>EHP-30<br>Baseline | 8 weeks	n=47<br>Mean age (SD)=36.43 (5.51)	Mean (SD)<br><br>Pain: 63.05 (12.27)<br>Control and Powerlessness: 60.57 (15.93)<br>Emotional Well-Being: 61.76 (12.56)<br>Social Support: 59.89 (13.41)<br>Self-Image: 35.45 (13.56)	**Vaginal Cream from Cymbopogon citratus Oil**<br><br>Mean difference p<br><br>Pain: p<0.001<br>Control and Powerlessness: p<0.001<br>Emotional Well-Being: p<0.001	None

Footnote: BMI = body mass index; CI = confidence interval; CPP = chronic pelvic pain; HRQoL = health-related quality of life; QoL = quality of life; MD = MD; NA = not answered; NRS = numeric rating scale; UK = United Kingdom; USA = United States of America; RCT = randomized controlled trial; SD = standard deviation; VAS = visual analogue scale. Bold is for significant associated factors.

Reference list:

Abokhrais, I., Denison, F., Whitaker, L., Saunders, P., Doust, A, Williams, L., Horne, A., Abokhrais, I.M., Denison, F.C., Whitaker, L.H.R., Saunders, P.T.K., Doust, Ann, Williams, L.J., Horne, A.W., 2020. A two-arm parallel double-blind randomised controlled pilot trial of the efficacy of Omega-3 polyunsaturated fatty acids for the treatment of women with endometriosis-associated pain (PurFECT1). PLoS One 15. https://doi.org/10.1371/journal.pone.0227695

Alberico D, Somigliana E, Bracco B, Dhouha D, Roberto A, Mosconi P, Facchin F, Vercellini P, 2018. Potential benefits of pregnancy on endometriosis symptoms. Eur J Obstet Gynecol Reprod Biol 230, 182–187. https://doi.org/10.1016/j.ejogrb.2018.08.576

Barberis N, Cannavò M, Cuzzocrea F, Saladino V, Verrastro V, 2023. “Illness perceptions and factors of distress as mediators between trait emotional intelligence and quality of life in endometriosis”. Psychol Health Med 28, 1818–1830. https://doi.org/10.1080/13548506.2023.2175878

Bi XL, Xie CX, 2018. Effect of neuromuscular electrical stimulation for endometriosis-associated pain: A retrospective study. Medicine (Baltimore) 97, e11266. https://doi.org/10.1097/MD.0000000000011266

Bień A, Rzońca E, Zarajczyk M, Wilkosz K, Wdowiak A, Iwanowicz-Palus G, 2020. Quality of life in women with endometriosis: a cross-sectional survey. Qual Life Res 29, 2669–2677. https://doi.org/10.1007/s11136-020-02515-4

Byrne, D., Curnow, T., Vashisht, A., Clark, T., Byrne, D.L., Curnow, T.L., Vashisht, A., Clark, T.J., BSGE Endometriosis Ctr, 2021. Analysis of factors that could affect symptomatic outcome in patients having laparoscopic excision of deep rectovaginal endometriosis in BSGE endometriosis centres. Eur. J. Obstet. Gynecol. Reprod. Biol. 261, 17–24. https://doi.org/10.1016/j.ejogrb.2021.04.007

Chen H, Vannuccini S, Capezzuoli T, Ceccaroni M, Mubiao L, Shuting H, Wu Y, Huang H, Petraglia F, 2021. Comorbidities and Quality of Life in Women Undergoing First Surgery for Endometriosis: Differences Between Chinese and Italian Population. Reprod Sci 28, 2359–2366. https://doi.org/10.1007/s43032-021-00487-5

Cofini V, Muselli M, Petrucci E, Lolli C, Pelaccia E, Guido M, Marinangeli F, Fabiani L, Necozione S, 2024. Factors associated with chronic pelvic pain in women with endometriosis: A national study on clinical and sociodemographic characteristics, lifestyles, quality of life, and perceptions of quality of care, during the COVID-19 pandemic. Womens Health (Lond) 20. https://doi.org/10.1177/17455057241227361

Daraï C, Deboute O, Zacharopoulou C, Laas E, Canlorbe G, Belghiti J, Zilberman S, Ballester M, Daraï E, 2015. Impact of osteopathic manipulative therapy on quality of life of patients with deep infiltrating endometriosis with colorectal involvement: results of a pilot study. Eur J Obstet Gynecol Reprod Biol 188, 70–3. https://doi.org/10.1016/j.ejogrb.2015.03.001

de Barros Meneguetti M, Silva FP, Dias GN, Benetti-Pinto CL, Angerame Yela D, 2023. Assessment of quality of life and psychological repercussions in women with endometriosis according to pain intensity. Psychol Health Med 28, 660–669. https://doi.org/10.1080/13548506.2022.2121972

de Freitas Fonseca M, Aragao LC, Sessa FV, Dutra de Resende JA Jr, Crispi CP, 2018. Interrelationships among endometriosis-related pain symptoms and their effects on health-related quality of life: a sectional observational study. Obstet Gynecol Sci 61, 605–614. https://doi.org/10.5468/ogs.2018.61.5.605

De Graaff AA, Dirksen CD, Simoens S, De Bie B, Hummelshoj L, D'Hooghe TM, Dunselman GA, 2015. Quality of life outcomes in women with endometriosis are highly influenced by recruitment strategies. Hum Reprod 30, 1331–41. https://doi.org/10.1093/humrep/dev084

De Hoyos G, Ramos-Sostre D, Torres-Reverón A, Barros-Cartagena B, López-Rodríguez V, Nieves-Vázquez C, Santiago-Saavedra F, Appleyard CB, Castro EM, Flores I, 2023. Efficacy of an environmental enrichment intervention for endometriosis: a pilot study. Front Psychol 14. https://doi.org/10.3389/fpsyg.2023.1225790

de Sousa, T., de Souza, B., Zomkowisk, K, da Rosa, P., Sperandio, F., de Sousa, T.R., de Souza, B.C., Zomkowisk, Kamilla, da Rosa, P.C., Sperandio, F.F., 2016. The effect of acupuncture on pain, dyspareunia, and quality of life in Brazilian women with endometriosis: A randomized clinical trial. Complement. Ther. Clin. Pract. 25, 114–121. https://doi.org/10.1016/j.ctcp.2016.09.006

Del Forno S, Raspollini A, Doglioli M, Andreotti A, Spagnolo E, Lenzi J, Borghese G, Raimondo D, Arena A, Rodriguez E, Hernandez A, Govoni F, Seracchioli R, 2024. Painful sexual intercourse, quality of life and sexual function in patients with endometriosis: not just deep dyspareunia. Arch Gynecol Obstet 310, 2091–2100. https://doi.org/10.1007/s00404-024-07643-7

Farshi N, Hasanpour S, Mirghafourvand M, Esmaeilpour K, 2020. Effect of self-care counselling on depression and anxiety in women with endometriosis: a randomized controlled trial. BMC Psychiatry 20, 391. https://doi.org/10.1186/s12888-020-02795-7

Flower A, Lewith GT, Little P, 2011. A feasibility study exploring the role of Chinese herbal medicine in the treatment of endometriosis. J Altern Complement Med 17, 691–9. https://doi.org/10.1089/acm.2010.0073

Fourquet J, Báez L, Figueroa M, Iriarte RI, Flores I, 2011. Quantification of the impact of endometriosis symptoms on health-related quality of life and work productivity. Fertil Steril 96, 107–12. https://doi.org/10.1016/j.fertnstert.2011.04.095

Friggi Sebe Petrelluzzi K, Garcia MC, Petta CA, Ribeiro DA, de Oliveira Monteiro NR, Céspedes IC, Spadari RC, 2012. Physical therapy and psychological intervention normalize cortisol levels and improve vitality in women with endometriosis. J Psychosom Obstet Gynaecol 33, 191–8. https://doi.org/10.3109/0167482X.2012.729625

Gioia F, Parola A, Boursier V, 2023. Alexithymia and Loneliness in Women with Endometriosis. Testing the Factorial Structure of the Italian Endometriosis Health Profile (EHP-30) and a Mediation Model. Clin Neuropsychiatry 20, 442–452. https://doi.org/10.36131/cnfioritieditore20230506

Gonçalves AV, Barros NF, Bahamondes L, 2017. The Practice of Hatha Yoga for the Treatment of Pain Associated with Endometriosis. J Altern Complement Med 23, 45–52. https://doi.org/10.1089/acm.2015.0343

González-Echevarría AM, Rosario E, Acevedo S, Flores I, 2019. Impact of coping strategies on quality of life of adolescents and young women with endometriosis. J Psychosom Obstet Gynaecol 40, 138–145. https://doi.org/10.1080/0167482X.2018.1450384

Grundström H, Engman L, Rimhagen E, Söderstierna C, Flink I, 2023. Experiences of communication in women with endometriosis: perceived validation and invalidation in different contexts, and associations with health-related quality of life. J Psychosom Obstet Gynaecol 44. https://doi.org/10.1080/0167482X.2023.2264483

Güvenç İB, Bozo Ö, 2023. Health-related quality of life of women with endometriosis: An Attachment-Diathesis Model of Chronic Pain perspective. Health Care Women Int 1–18. https://doi.org/10.1080/07399332.2023.2177295

Hansen, K., Kesmodel, U., Kold, M, Forman, A, Hansen, K.E., Kesmodel, U.S., Kold, Mette, Forman, Axel, 2017. Long-term effects of mindfulness-based psychological intervention for coping with pain in endometriosis: A six-year follow-up on a pilot study. Nord. Psychol. 69, 100–109. https://doi.org/10.1080/19012276.2016.1181562

Khan R, Baksh R, Wallace TJ, Aimable M, Bujhawan V, Cumberbatch J, Marbella EJ, Phagoo D, Ramjattan S, Shavili A, 2024. A cross-sectional study on the quality of life of women with endometriosis in Trinidad and Tobago. Front Glob Womens Health 5. https://doi.org/10.3389/fgwh.2024.1359741

Kiykac Altinbas S, Bayoglu Tekin Y, Dilbaz B, Dilbaz S, 2015. Evaluation of quality of life in fertile Turkish women with severe endometriosis. J Obstet Gynaecol 35, 49–52. https://doi.org/10.3109/01443615.2014.930110

Kold, M, Hansen, T, Vedsted-Hansen, H, Forman, A, Kold, Mette, Hansen, Tia, Vedsted-Hansen, Hanne, Forman, Axel, 2012. Mindfulness-based psychological intervention for coping with pain in endometriosis. Nord. Psychol. 64, 2–16. https://doi.org/10.1080/19012276.2012.693727

Krabbenborg I, de Roos N, van der Grinten P, Nap A, 2021. Diet quality and perceived effects of dietary changes in Dutch endometriosis patients: an observational study. Reprod Biomed Online 43, 952–961. https://doi.org/10.1016/j.rbmo.2021.07.011

Márki G, Bokor A, Rigó J, Rigó A, 2017. Physical pain and emotion regulation as the main predictive factors of health-related quality of life in women living with endometriosis. Hum Reprod 32, 1432–1438. https://doi.org/10.1093/humrep/dex091

Martins, J., Ferreira, G., Vilaca, M., Ferreira, H., Osorio, F., Nogueira-Silva, C., Pereira, M., Martins, J., Ferreira, G., Vilaca, M., Ferreira, H., Osorio, F., Nogueira-Silva, C., Pereira, M.G., 2022. Quality of life and sexual satisfaction in women with endometriosis: the moderator role of symptom severity. Psychol. Sex. 13, 952–964. https://doi.org/10.1080/19419899.2021.1943501

Matasariu RD, Mihaila A, Iacob M, Dumitrascu I, Onofriescu M, Crumpei Tanasa I, Vulpoi C, 2017. Psycho-social aspects of quality of life in women with endometriosis. Acta Endocrinol (Buchar) 13, 334–339. https://doi.org/10.4183/aeb.2017.334

Matías-González Y, Sánchez-Galarza A, Rosario-Hernández E, Flores-Caldera I, Rivera-Segarra E, 2022. Stigma and social support and their impact on quality of life and self-esteem among women with endometriosis in Latin-America and the Caribbean. PLOS Glob Public Health 2, e0001329. https://doi.org/10.1371/journal.pgph.0001329

Meissner K, Schweizer-Arau A, Limmer A, Preibisch C, Popovici RM, Lange I, de Oriol B, Beissner F, 2016. Psychotherapy With Somatosensory Stimulation for Endometriosis-Associated Pain: A Randomized Controlled Trial. Obstet Gynecol 128, 1134–1142. https://doi.org/10.1097/AOG.0000000000001691

Merlot B, Elie V, Périgord A, Husson Z, Jubert A, Chanavaz-Lacheray I, Dennis T, Cotty-Eslous M, Roman H, 2023. Pain Reduction With an Immersive Digital Therapeutic in Women Living With Endometriosis-Related Pelvic Pain: At-Home Self-Administered Randomized Controlled Trial. J Med Internet Res 25, e47869. https://doi.org/10.2196/47869

Miazga E, Starkman H, Schroeder N, Nensi A, McCaffrey C, 2024. Virtual Mindfulness-Based Therapy for the Management of Endometriosis Chronic Pelvic Pain: A Novel Delivery Platform to Increase Access to Care. J Obstet Gynaecol Can 46. https://doi.org/10.1016/j.jogc.2024.102457

Mira TAA, Yela DA, Podgaec S, Baracat EC, Benetti-Pinto CL, 2020. Hormonal treatment isolated versus hormonal treatment associated with electrotherapy for pelvic pain control in deep endometriosis: Randomized clinical trial. Eur J Obstet Gynecol Reprod Biol 255, 134–141. https://doi.org/10.1016/j.ejogrb.2020.10.018

Montanari G, Di Donato N, Benfenati A, Giovanardi G, Zannoni L, Vicenzi C, Solfrini S, Mignemi G, Villa G, Mabrouk M, Schioppa C, Venturoli S, Seracchioli R, 2013. Women with deep infiltrating endometriosis: sexual satisfaction, desire, orgasm, and pelvic problem interference with sex. J Sex Med 10, 1559–66. https://doi.org/10.1111/jsm.12133

Muselli M, Mancinelli M, Limoncin E, Lolli C, Pelaccia E, Guido M, Fabiani L, Necozione S, Cofini V, 2024. Investigating Unhealthy Behaviors Associated with SF-36 Domains in Women with Endometriosis-Findings from a Web-Based Survey Data Set. Behav Sci (Basel) 14. https://doi.org/10.3390/bs14030199

Norman M, Razmpour O, Olsen JM, 2021. Women’s Use of Self-Care Interventions for Endometriosis Pain in the United States. Nurs Womens Health 25, 346–356. https://doi.org/10.1016/j.nwh.2021.07.008

Pessoa de Farias Rodrigues M, Lima Vilarino F, de Souza Barbeiro Munhoz A, da Silva Paiva L, de Alcantara Sousa LV, Zaia V, Parente Barbosa C, 2020. Clinical aspects and the quality of life among women with endometriosis and infertility: a cross-sectional study. BMC Womens Health 20, 124. https://doi.org/10.1186/s12905-020-00987-7

Pinot-Monange A, Moisset X, Chauvet P, Gremeau AS, Comptour A, Canis M, Pereira B, Bourdel N, 2019. Repetitive Transcranial Magnetic Stimulation Therapy (rTMS) for Endometriosis Patients with Refractory Pelvic Chronic Pain: A Pilot Study. J Clin Med 8. https://doi.org/10.3390/jcm8040508

Pontoppidan, K., Olovsson, M., Grundstrom, H., 2023. Clinical factors associated with quality of life among women with endometriosis: a cross-sectional study. BMC WOMENS HEALTH 23. https://doi.org/10.1186/s12905-023-02694-5

Ravins I, Joseph G, Tene L, 2023. The Effect of Practicing “Endometriosis Yoga” on Stress and Quality of Life for Women with Endometriosis: AB Design Pilot Study. Altern Ther Health Med 29, 8–14.

Rees M, Kiemle G, Slade P, 2022. Psychological variables and quality of life in women with endometriosis. J Psychosom Obstet Gynaecol 43, 58–65. https://doi.org/10.1080/0167482X.2020.1784874

Rohloff N, Götz T, Kortekamp SS, Heinze NR, Weber C, Schäfer SD, 2024a. Influence of App-Based Self-Management on the Quality of Life of Women With Endometriosis. Cureus 16. https://doi.org/10.7759/cureus.67655

Rohloff N, Rothenhöfer M, Götz T, Schäfer SD, 2024b. Observational pilot study on the influence of an app-based self-management program on the quality of life of women with endometriosis. Arch Gynecol Obstet 310, 1157–1170. https://doi.org/10.1007/s00404-024-07468-4

Sepulcri Rde P, do Amaral VF, 2009. Depressive symptoms, anxiety, and quality of life in women with pelvic endometriosis. Eur J Obstet Gynecol Reprod Biol 142, 53–6. https://doi.org/10.1016/j.ejogrb.2008.09.003

Sesti F, Pietropolli A, Capozzolo T, Broccoli P, Pierangeli S, Bollea MR, Piccione E, 2007. Hormonal suppression treatment or dietary therapy versus placebo in the control of painful symptoms after conservative surgery for endometriosis stage III-IV. A randomized comparative trial. Fertil Steril 88, 1541–7. https://doi.org/10.1016/j.fertnstert.2007.01.053

Simonsen, S.M., Strømberg, C., Zoffmann, V., Hartwell, D., Olesen, M.L., 2020. About me as a person not only the disease – piloting Guided Self-Determination in an outpatient endometriosis setting. Scandinavian Caring Sciences 34, 1017–1027. https://doi.org/10.1111/scs.12810

Škegro B, Bjedov S, Mikuš M, Mustač F, Lešin J, Matijević V, Ćorić M, Elveđi Gašparović V, Medić F, Sokol Karadjole V, 2021. Endometriosis, Pain and Mental Health. Psychiatr Danub 33, 632–636.

Skinner CM, Kuijer RG, 2024. Self-compassion and health-related quality of life in individuals with endometriosis. Psychol Health 1–18. https://doi.org/10.1080/08870446.2024.2325506

Soliman AM, Coyne KS, Zaiser E, Castelli-Haley J, Fuldeore MJ, 2017. The burden of endometriosis symptoms on health-related quality of life in women in the United States: a cross-sectional study. J Psychosom Obstet Gynaecol 38, 238–248. https://doi.org/10.1080/0167482X.2017.1289512

Spinoni M, Capano AU, Porpora MG, Grano C, 2024. Understanding the Psychological Factors Linking Pelvic Pain and Health-related Quality of Life in Endometriosis: The Influence of Illness Representations and Coping Strategies. Am J Obstet Gynecol. https://doi.org/10.1016/j.ajog.2024.12.027

Stochino Loi E, Pontis A, Cofelice V, Pirarba S, Fais MF, Daniilidis A, Melis I, Paoletti AM, Angioni S, 2019. Effect of ultramicronized-palmitoylethanolamide and co-micronized palmitoylethanolamide/polydatin on chronic pelvic pain and quality of life in endometriosis patients: An open-label pilot study. Int J Womens Health 11, 443–449. https://doi.org/10.2147/IJWH.S204275

Sullivan-Myers C, Sherman KA, Beath AP, Duckworth TJ, Cooper MJW, 2021. Delineating sociodemographic, medical and quality of life factors associated with psychological distress in individuals with endometriosis. Hum Reprod 36, 2170–2180. https://doi.org/10.1093/humrep/deab138

Thabet AAE, Alshehri MA, 2018. Effect of Pulsed High-Intensity Laser Therapy on Pain, Adhesions, and Quality of Life in Women Having Endometriosis: A Randomized Controlled Trial. Photomed Laser Surg 36, 363–369. https://doi.org/10.1089/pho.2017.4419

Thammasiri, C, Amnatbuddee, S, Sothornwit, J, Temtanakitpaisan, T, Buppasiri, P, Thammasiri, Chutikarn, Amnatbuddee, Siriruthai, Sothornwit, Jen, Temtanakitpaisan, Teerayut, Buppasiri, Pranom, 2022. A Cross-Sectional Study on the Quality of Life in Women with Endometrioma. Int. J. Womens Health 14, 9–14. https://doi.org/10.2147/IJWH.S341603

Touboul C, Amate P, Ballester M, Bazot M, Fauconnier A, Daraï E, 2013. Quality of Life Assessment Using EuroQOL EQ-5D Questionnaire in Patients with Deep Infiltrating Endometriosis: The Relation with Symptoms and Locations. Int J Chronic Dis 2013, 452134. https://doi.org/10.1155/2013/452134

van Haaps, A.P., Wijbers, V., J., Schreurs, A.M.F., Vlek, S., Tuynman, J., De Bie, B., de Vogel, A.L., van Wely, M., Mijatovic, V., 2023. The effect of dietary interventions on pain and quality of life in women diagnosed with endometriosis: a prospective study with control group. HUMAN REPRODUCTION 38, 2433–2446. https://doi.org/10.1093/humrep/dead214

Van Niekerk L, Johnstone L, Matthewson M, 2022. Health-related quality of life in endometriosis: The influence of endometriosis-related symptom presence and distress. J Health Psychol 27, 3121–3135. https://doi.org/10.1177/13591053221085051

Van Niekerk LM, Dell B, Johnstone L, Matthewson M, Quinn M, 2023. Examining the associations between self and body compassion and health related quality of life in people diagnosed with endometriosis. J Psychosom Res 167, 111202. https://doi.org/10.1016/j.jpsychores.2023.111202

Wu YH, Lu YY, Liu KF, 2024. Factors influencing health-related quality of life in women with endometriosis: A cross-sectional study. Nurs Health Sci 26. https://doi.org/10.1111/nhs.13100

Yazdankhah, Z., Aminimoghaddam, S., Rahimi, R., Kheiri, S., Bioos, S., Tansaz, M., Sadeghi, S., 2024. Efficacy of a Vaginal Cream from Cymbopogon citratus Oil in Patients with Endometrioma: a Single-Arm Clinical Trial. RESEARCH JOURNAL OF PHARMACOGNOSY 12, 21–29. https://doi.org/10.22127/rjp.2024.478866.2605

Supplementary Table 3: Summary of included studies

**Author, year, country**	**Design / Purpose**	**Intervention or exposure potentially associated with QoL / QoL score and data point**	**Participants (n, age)**	**Level of quality of life at baseline**	**Factors significantly associated with improved quality of life**	**Factors significantly associated with a deterioration in quality of life**
Apers et al., 2018 Netherlan	Cross sectional<br><br>Investigate the	Exposure: Patient-centred endometriosis care (PCEC) measured	n=109<br><br>Mean age (SD) =	Mean (SD) = 29.3 (23.7)<br><br>Item:	Regression analysis: **PCEC-subscale "continuity"** (B=0.27, p=0.029).	None

Author, year, country	Design / Purpose	Intervention or exposure potentially associated with QoL / QoL score and data point	Participants (n, age)	Level of quality of life at baseline	Factors significantly associated with improved quality of life	Factors significantly associated with a deterioration in quality of life
ds	association between patient-centered endometriosis care (PCEC) and HRQoL.	with ENDOCARE questionnaire (continuous)<br><br>Control: None<br><br>EHP-30<br>One data point	35.4 (5.3)	Self image: 24.4 (26.5)<br>Control: 35.9 (30.4)<br>Pain: 25.6 (25.8)<br>Emotional: 31.2 (25.3)<br>Social: 30.6 (27.5)	Correlation analysis:<br>**PCEC-subscale "information, communication and education"** (r=0.21, p=0.033)<br><br>**PCEC-subscale "continuity and transition"** (r=0.29, p=0.008)<br><br>**PCEC-subscale "respect for patients' values, preferences and expressed needs"** (r=0.19, p=0.046)	
Artacho-Cordón et al., 2023 Spain	RCT<br><br>Evaluate the effectiveness of "Physio-EndEA" on quality of life, pain, and lumbopelvic impairments in women with endometriosis unresponsive to conventional therapy.	Intervention: Physio-EndEA, a multimodal nine-week supervised exercise intervention<br><br>Control: Usual treatment<br><br>EHP-30<br>Baseline | nine weeks | 1 year	Intervention: n=16<br>Mean age (SD) = 36.31 (6.28)<br><br>Control: n=15<br>Mean age (SD) = 38.40 (4.19)	Mean (SD)<br><br>Intervention: 50.04 (18.05)<br>Control: 56.84 (15.35)	**Multimodal supervised exercise intervention**<br><br>Mean difference between-group adjusted (CI):<br>Baseline-post intervention: -9.08 (-17.38,-0.30)<br>Baseline-1year: -11.08 (-18.49,-3.66)	None
Bień et al., 2024 Poland	Cross sectional<br><br>Analyse selected clinical data affecting the quality of life of women with endometriosis.	Exposure:<br>- Average duration of menstrual bleeding (continuous)<br>- Occurrence of regular menstruation (dichotomous)<br>- Occurence of heavy menstruation	n=425<br><br>Mean age (SD) = 31.07 (6.45)	Mean (SD) = 30.21 (26.65)	**Fewer heavy periods:** $\beta = -0.141$; $p = 0.006$	**Painful periods:** $\beta = 0.111$; $p = 0.030$<br><br>**Pain during intercourse:** $\beta = 0.192$; $p < 0.001$

**Author, year, country**	**Design / Purpose**	**Intervention or exposure potentially associated with QoL / QoL score and data point**	**Participants (n, age)**	**Level of quality of life at baseline**	**Factors significantly associated with improved quality of life**	**Factors significantly associated with a deterioration in quality of life**
		(dichotomous)<br>- Painful menstruation (dichotomous)<br>- Pain during the cycle (dichotomous)<br>- Dyspareunia (dichotomous or not concerned)<br><br>Control: None<br><br>EHP-30<br>One data point				
Breton et al., 2025 France	Cohort study<br><br>Measure the impact of a digital health program on the symptoms and quality of life levels of women with endometriosis.	Intervention: Digital health program providing medical and scientific information about endometriosis and multidisciplinary self-management tools<br><br>Control: Women with endometriosis who did not follow the program<br><br>EHP-5 and EQ-5D<br>Baseline | 3 months	Intervention:<br>n=92<br>Mean age (SD) = 36.7 (6.8)<br><br>Control:<br>n=149<br>Mean age (SD) = 36.6 (7.2)	Intervention:<br>EHP-5 Mean (SD) = 53.8 (20.4)<br>EQ-5D Mean (SD) = 0.8 (0.1)<br><br>Control:<br>EHP-5 Mean (SD) = 45.2 (20.3)<br>EQ-5D Mean (SD) = 0.8 (0.2)	**Digital health program**<br>Mean difference (SD):<br><br>EHP-5<br>Intervention: -5.9 (21.0)<br>Control: 1.0 (14.8)<br>p=0.03<br><br>EQ-5D<br>Intervention: 0.1 (0.1)<br>Control: -0.0 (0.1)<br>p=0.001	None
Cofini et al., 2023 Italy	Cross sectional<br><br>Examine the QoL in women with endometriosis and its relationship to	Exposure:<br>- Quality of Care (QoC) Perception (satisfaction index - continuous)<br>- Age (continuous)<br>- Occupational status (dichotomous)	n=875<br><br>Mean age (SD) = 35 (8)	Mean (SD)<br><br>Physical: 38.89 (10.55)<br>Mental: 34.59 (11.17)	**Have physical activity:**<br>Physical: $\beta=2.0$ $p=0.003$<br>Mental: $\beta=1.9$ $p=0.018$<br><br>**Satisfaction index (quality of care):**<br>Physical: $\beta=0.4$ $p<0.001$<br>Mental: $\beta=0.4$ $p<0.001$	**Smoking:**<br>Physical: $\beta=-2.3$ $p=0.006$<br>Mental: $\beta=-2.3$ $p=0.016$<br><br>**Comorbidities:**<br>Physical: $\beta=-5.0$ $p<0.001$<br>Mental: $\beta=-1.6$ $p=0.036$

Author, year, country	Design / Purpose	Intervention or exposure potentially associated with QoL / QoL score and data point	Participants (n, age)	Level of quality of life at baseline	Factors significantly associated with improved quality of life	Factors significantly associated with a deterioration in quality of life
	the quality of care.	- Educational level (high/low)<br>- BMI (continuous)<br>- Physical activity (dichotomous)<br>- Smoker (dichotomous)<br>- Alcohol consumption (dichotomous)<br>- Full-term pregnancy (dichotomous)<br>- Comorbidities (dichotomous)<br><br>Control: None<br><br>SF-36<br>One data point			**Older age:**<br>Mental: β=0.2 p=0.012<br><br>**Employed:**<br>Physical: β=2.3 p=0.002<br><br>**High educational level:**<br>Physical: β=3.2 p<0.001	**Alcohol use:**<br>Physical: β=2.0 p=0.009<br><br>**Full term pregnancy:**<br>Physical: β=−2.5 p=0.003
Comptour et al., 2024 France	Cross sectional<br><br>Investigate the association between the severity of endometriosis and age at initial diagnosis.	Exposure:<br>- Endometriosis staging (rAFS)<br>- Age (5 categories)<br><br>Control: None<br><br>SF-36<br>One data point	n=964<br><br>Age (n):<br><=25yo: 73<br>26-30yo: 240<br>31-35yo: 282<br>36-40yo: 181<br>>40yo: 139	Mean (SD)<br><br>Physical:<br><=25yo: 48.0 (9.3)<br>26-30yo: 48.1 (10.3)<br>31-35yo: 48.9 (10.7)<br>36-40yo: 48.3 (10.4)<br>>40yo: 47.4 (11.2)<br><br>Mental:<br><=25yo: 40 (11.5)<br>26-30yo: 41.2 (13)<br>31-35yo: 42.5 (12.6)<br>36-40yo: 40.5 (11.2)<br>>40yo: 44.1 (13.4)	None	None
Donatti et al., 2024 Brazil	RCT<br><br>Assess the efficacy of	Intervention: CBT, 16 sessions, one per week, 4 months	Intervention:<br>n=25<br>Mean age: 36	Approximative Mean:<br><br>Intervention:<br>Physical wellbeing: 60	**CBT**<br><br>Estimated difference of improvements and p-value	None

Author, year, country	Design / Purpose	Intervention or exposure potentially associated with QoL / QoL score and data point	Participants (n, age)	Level of quality of life at baseline	Factors significantly associated with improved quality of life	Factors significantly associated with a deterioration in quality of life
	Cognitive-Behavioral Therapy (CBT) in enhancing coping strategies, alleviating depression, stress, pain perception, and improving the quality of life for women with endometriosis.	Control: Standard care<br><br>SF-36<br>Baseline | 4 months	Control:<br>n=27<br>Mean age: 32	Physical role limitations: 25<br>Bodily Pain: 40<br>General: 40<br>Fatigue/Vitality: 40<br>Social: 50<br>Emotional role limitations: 10<br>Emotional well-being:50<br><br>Control:<br>Physical wellbeing:60<br>Physical role limitations: 25<br>Bodily Pain: 30<br>General: 40<br>Fatigue/Vitality: 30<br>Social: 40<br>Emotional role limitations: 30<br>Emotional well-being: 40	intervention vs. control:<br><br>Physical wellbeing: 8.20 p=0.14<br>Physical role limitations: 26.89 p=0.02<br>Bodily Pain: 13.84 p=0.01<br>General: 23.32 p<0.01<br>Fatigue/Vitality: 29.00 p<0.01<br>Social: 33.07 p<0.01<br>Emotional role limitations: 52.84 p<0.01<br>Emotional well-being: 30.41 p<0.01	
Gudarzi et al., 2023 Iran	RCT<br><br>Determine the effect of curcumin on painful symptoms of endometriosis and the quality of life in affected women.	Intervention: Curcumin (500mg, 2/day, 8 weeks)<br><br>Control: Placebo<br><br>EHP-30<br>Baseline | 8 weeks	n=68<br><br>Intervention:<br>n=34<br>Mean age (SD)=33.5 (5.7)<br><br>Control:<br>n=34<br>Mean age (SD)=32.1 (4.8)	Mean (SD)<br><br>Intervention: 33.8 (18.7)<br><br>Control: 30.0 (13.2)	None	None
Hansen et al., 2023 Denmark	RCT<br><br>Evaluate the effect of psychological intervention on	Intervention:<br>- Specific mindfulness- and acceptance-based psychological intervention (MY-ENDO), 10-week	MY-ENDO:<br>n=20<br>Mean age (SD) = 28.95 (7.84)<br><br>Non-specific	Mean (SD)<br><br>MY-ENDO:<br>Pain: 51.95 (20.6)<br>Control and powerlessness: 68.15 (17.27)	**Psychological intervention vs waitlist:**<br>Control and powerlessness: Cohen's d=0.78 F=6.03 P=0.019<br>Emotional wellbeing: Cohen's	None

Author, year, country	Design / Purpose	Intervention or exposure potentially associated with QoL / QoL score and data point	Participants (n, age)	Level of quality of life at baseline	Factors significantly associated with improved quality of life	Factors significantly associated with a deterioration in quality of life
	CPP and QoL in women with endometriosis.	program including 3-h weekly group sessions<br>- Non-specific psychological intervention<br><br>Control: Standard medical treatment, waitlist<br><br>EHP-30<br>Baseline | Post-intervention	intervention:<br>n=19<br>Mean age (SD) = 33.84 (7.69)<br><br>Control:<br>n=19<br>Mean age (SD) = 32.81 (9.01)	Emotional wellbeing: 47.32 (10.92)<br>Social support: 51.34 (26.08)<br>Self-image: 51.79 (29.63)<br><br>Non-specific intervention:<br>Pain: 47.42 (14.45)<br>Control and powerlessness: 62.78 (15.14)<br>Emotional wellbeing: 42.78 (15.39)<br>Social support: 48.75 (27.77)<br>Self-image: 55.56 (23.92)<br><br>Control:<br>Pain: 47.20 (19.99)<br>Control and powerlessness: 60.26 (25.15)<br>Emotional wellbeing: 47.44 (22.79)<br>Social support: 53.85 (24.94)<br>Self-image: 49.36 (23.93)	d=1.01 F=10.17 P=0.003<br>Social support: Cohen's d=0.66 F=4.41 P=0.042	
He et al., 2022 China	Cross sectional<br><br>Investigate the influence of QOL on unhealthy emotions as well as relevant factors among patients with endometriosis.	Exposure:<br>- Anxiety - Hamilton Anxiety Inventory (HAMA - 3 categories)<br>- Depression - Depression Anxiety Scale (SDS - 3 categories)<br><br>Control: None<br><br>SF-12	n=139<br><br>Mean age (SD) = 35.8 (6.79)	Mean (SD)<br><br>Physical: 42.0 (8.99)<br>Mental: 44.0 (11.91)	NA	**Anxiety:**<br>Physical: OR = 0.942, 95%CI: 0.97–0.977<br>Mental: OR = 0.905, 95%CI: 0.877–0.934<br><br>**Depression:**<br>Mental: OR = 0.899, 95%CI: 0.866–0.933

Author, year, country	Design / Purpose	Intervention or exposure potentially associated with QoL / QoL score and data point	Participants (n, age)	Level of quality of life at baseline	Factors significantly associated with improved quality of life	Factors significantly associated with a deterioration in quality of life
		One date point				
Kanti, Allard, Maheux-Lacroix et al., 2024 Canada	Cross sectional<br><br>Investigate the quality of life and pain scores of patients with endometriomas compared to those with other types of endometriosis lesions.	Exposure: Endometrioma<br><br>Control: Other endometriosis lesions<br><br>EHP-30<br>One data point	n=248<br>Mean age (SD)=37.1 (7.5)<br><br>Exposure:<br>n=81<br>Mean age (SD)=37.0 (7.1)<br><br>Control:<br>n=167<br>Mean age (SD)=37.1 (7.8)	Mean (SD)<br><br>Overall: 45.9 (25.9)<br><br>Exposure: 42.4 (25.2)<br><br>Control: 47.6 (26.2)	None	None
Kanti, Allard, Métivier et al., 2024 Canada	Cross sectional<br><br>Identify pain phenotypes in patients with endometriosis and investigate their associations with demographics, clinical characteristics, comorbidities and pain-related quality of life.	Exposure: More severe and frequent symptoms<br><br>Control: Mild and less frequent symptoms<br><br>EHP-30<br>One data point	n=352<br>Mean age (SD)=36 (7)<br><br>Exposure:<br>n=189<br><br>Control:<br>n=163	Mean (CI)<br><br>Exposure: 59 (56-62)<br><br>Control: 33 (29-37)	None	Mean difference p<br><br>**More severe and frequent symptoms**<br>vs. mild and less frequent symptoms<br>p<0.001
Li et al., 2023 China	RCT<br><br>Evaluate the efficacy and safety of acupuncture in the treatment of	Intervention: Acupuncture - started 1 week before the expected onset of menstruation of each subject and was administered in 30-	Intervention:<br>n=53<br>Mean age (SD) = 30.3 (4.6)<br><br>Control:<br>n=53	Mean (SD)<br><br>Intervention: 104.2 (35.2)<br><br>Control: 103.0 (32.6)	**Acupuncture**<br>Change between baseline and week 12 in acupuncture vs sham: MD=-18.84 CI=(-29.33, -8.36) p=0.0005	None

Author, year, country	Design / Purpose	Intervention or exposure potentially associated with QoL / QoL score and data point	Participants (n, age)	Level of quality of life at baseline	Factors significantly associated with improved quality of life	Factors significantly associated with a deterioration in quality of life
	endometriosis-associated pain.	minute sessions once daily, 3 times a week.<br><br>Control: Sham acupuncture<br><br>EHP-30 (0-212)<br>Baseline | 12 weeks | 24 weeks	Mean age (SD) = 30.1 (4.5)			
McPeak et al., 2022 Canada	Cross sectional<br><br>Determine if pain catastrophizing is independently associated with pain HRQoL in women with endometriosis, independent of potential confounders.	Exposure:<br>- Pain Catastrophizing Score (continuous)<br>- Age (continuous)<br>- Pain: CPP, dysmenorrhea, abdominal wall pain, back pain (0-10 - continuous)<br><br>Control: None<br><br>EHP-30<br>One data point	n=236<br><br>Mean age (SD) = 35.0 (7.3)	No data	**Age:**<br>β =-0.13 p=0.039	**Higher score on Pain Catastrophizing Scale:**<br>B=0.52 p< 0.001<br><br>**More severe CPP:**<br>B=1.40 p< 0.001<br><br>**More severe dysmenorrhea:**<br>B=2.82 p< 0.001<br><br>**Presence of abdominal wall pain:**<br>B=4.31 p=0.029<br><br>**More severe back pain:**<br>B=0.72 p=0.034
Muharam et al., 2022 Indonesia	Cross sectional<br><br>Evaluate the association between pain characteristic, psychiatric disorder, and QoL in women with endometriosis.	Exposure:<br>- BMI (2 categories)<br>- Pain (VAS +/- 7)<br>- Psychiatric comorbidity (Mini-ICD - dichotomous)<br><br>Control: None<br><br>EHP-30<br>One data point	n=160<br><br>Mean age (SD) = NA	Mean (SD) = 39.05 (14.74)	None	**Increased pain:**<br>OR=13.33 (CI 9.01–17.65) p<0.001<br><br>**Presence of psychiatric disorder:**<br>OR=9.84 (CI 6.07–13.60) p<0.001<br><br>**Overweight BMI:**<br>OR=5.52 (CI 1.82–9.27), p=0.007

Author, year, country	Design / Purpose	Intervention or exposure potentially associated with QoL / QoL score and data point	Participants (n, age)	Level of quality of life at baseline	Factors significantly associated with improved quality of life	Factors significantly associated with a deterioration in quality of life
Mundo-López et al., 2020 Spain	Cross sectional<br><br>Analyze the levels of chronic fatigue in Spanish women with endometriosis and its relationship with their psychosocial status and QoL.	Exposure:<br>- Chronic fatigue (PFS - 3 categories)<br>- Pain (3 categories)<br>- Mood assessment (EVEA)<br>- Anxiety and depression (HADS - 2 categories)<br>- Pain Catastrophizing (PCS - continuous)<br>- Sleep Quality (PSQI - 2 categories)<br>- Sexual Function (FSFI - 2 categories)<br>- Gastrointestinal Quality of Life (GIQLI - continuous)<br>- Social Support (MOS-SSS - continuous)<br><br>Control: None<br><br>EHP-30<br>One data point	n = 230<br><br>Mean age (SD) = 36.7 (5.2)	Mean (SD) = 55.0 (1.7)	None	**Severe pain: β=**1.39 (CI 1.15-1.68), p=0.001<br><br>**Severe chronic fatigue: β=**1.56 (CI 1.28-1.91), p<0.001<br><br>**Poorer Gastrointestinal QoL: β=**1.83 (CI 1.51-2.23), p<0.001<br><br>**Poorer sexual function: β=**1.83 (CI 1.51-2.23), p<0.001<br><br>**Anxiety: β=**1.29 (CI 1.15-1.46) p<0.001<br><br>**Depression: β=**1.21 (CI 1.09-1.34) p<0.001<br><br>**Anger/hostility:** β=1.08 (CI 1.01-1.15), p=0.031<br><br>**Catastrophizing thoughts:** β=1.21 (CI 1.09-1.34) p<0.001
Muñoz-Gómez et al., 2023 Spain	RCT<br><br>To assess the effectiveness of a manual therapy protocol in terms of the clinical characteristics, QoL, and emotional	Intervention: Manual therapy protocol - 8 weeks, with one session for 30 min every 15 days<br><br>Control: Placebo group who received a hands-on placebo intervention<br><br>EHP-30 | SF-36	Intervention: n=21<br>Mean age (SD) = 34.85 (7.23)<br><br>Control: n=20<br>Mean age (SD) = 37.40 (6.62)	Mean (SD)<br><br>Intervention:<br>EHP-30 = 51.69 (16.01)<br>SF-36 = 51.28 (20.12)<br><br>Control:<br>EHP-30 = 46.81 (14.76)<br>SF-36 = 54.04 (20.23)	**Manual therapy protocol** (8 weeks):<br>Pain item: p=0.003 d=0.98	None

**Author, year, country**	**Design / Purpose**	**Intervention or exposure potentially associated with QoL / QoL score and data point**	**Participants (n, age)**	**Level of quality of life at baseline**	**Factors significantly associated with improved quality of life**	**Factors significantly associated with a deterioration in quality of life**
	condition of the women with endometriosis-related pelvic pain.	Baseline | Post-intervention | 1-month follow-up | 6-month follow-up				
Nodler et al., 2020 USA	RCT<br><br>Determine whether supplementation with vitamin D or ω-3 fatty acids remediates pain, changes frequency of pain medication usage, or affects QoL in young women with endometriosis.	Intervention:<br>- Supplementation with vitamin D, 2000 IU vitamin D3, daily for 6 months<br>- Supplementation with ω-3 fatty acids, 1000 mg fish oil, daily for 6 months<br><br>Control: Placebo group, daily for 6 months<br><br>SF-12<br>Baseline | 3-month | 6-month	Vitamin D:<br>n=23<br>Mean age (SD) = 20.0 (2.7)<br><br>Fish oil:<br>n=17<br>Mean age (SD) = 18.9 (3.1)<br><br>Control:<br>n=19<br>Mean age (SD) = 20.1 (3.5)	Mean (95CI)<br><br>Vitamin D:<br>Physical: 41.6 (CI=38.6 ; 44.7)<br>Mental: 44.5 (CI=40.1 ; 48.8)<br><br>Fish oil:<br>Physical: 45.7 (CI=42.0;49.5)<br>Mental: 44.1 (CI=39.0;49.2)<br><br>Control:<br>Physical: 46.8 (43.3;50.2)<br>Mental: 41.2 (CI=36.3;46.0)	None	None
O'Hara et al., 2021 Australia	Cross sectional<br><br>Identify the self-management factors associated with QoL among women with endometriosis.	Exposure:<br>- Age (continuous)<br>- Health insurance (dichotomous)<br>- Income perception (2 categories)<br>- University education (dichotomous)<br>- Pain severity (0-10 - continuous)<br>- Complementary practitioners (dichotomous)<br>- Chronic Disease Management plan	n=620<br><br>Mean age (SD) = 34.6 (9.5)	Mean (SD)<br><br>Physical: 42.9 (9.2)<br>Mental: 36.8 (11.3)	**Greater self-efficacy**<br>Physical: β=1.308 (CI=0.910;1.705) P<0.001<br>Mental: β=1.550 (1.039;2.061) P<0.001<br><br>**Income sufficiency**<br>Physical: β=3.309 (CI=1.888;4.729) P<0.001<br><br>**Being older**<br>Mental: β=0.185 (0.085;0.286) P<0.001<br><br>**Being partnered**	**Being older**<br>Physical: β=-0.149 (-0.228;-0.071) P<0.001<br><br>**Pain severity**<br>Physical: β=-0.615 (-0.917;-0.312) P<0.001<br><br>**Having a chronic disease management plan**<br>Physical: β=-2.607 (-4.330;-0.883) P<0.05<br><br>**Number of self-care activities**<br>Physical: β=-0.410 (-0.687;-

**Author, year, country**	**Design / Purpose**	**Intervention or exposure potentially associated with QoL / QoL score and data point**	**Participants (n, age)**	**Level of quality of life at baseline**	**Factors significantly associated with improved quality of life**	**Factors significantly associated with a deterioration in quality of life**
		(dichotomous)<br>- Self-efficacy score (continuous)<br>- Partners in health (continuous)<br>- Self-care activities (continuous)<br><br>Control: None<br><br>SF-36<br>One data point			Mental: β=3.512 (1.554;5.470) P<0.001<br><br>**University education**<br>Mental: β=1.832 (0.081;3.583) P<0.05<br><br>**Higher partners in health scores**<br>Mental: β=0.162 (0.084;0.240) P<0.001	0.133) P<0.05
Rodríguez-Ruiz et al., 2024<br>Spain	RCT<br><br>Evaluate the effectiveness of an integral HAMMAM experience, applied in a multisensorial immersive environment, on pain, well-being and quality of life (QoL) in women with endometriosis-related chronic pelvic pain that is unresponsive to conventional treatment.	Intervention: integral HAMMAM experience, a 4-week therapeutic program that combined hydrotherapy and Swedish massage, three sessions of 1.5 h each, separated by an interval of 14 days<br><br>Control: Standard care<br><br>EHP-30<br>Baseline | 4 weeks	n=44<br><br>Intervention:<br>n=21<br>Mean age (SD)=35.33 (6.65)<br><br>Control:<br>n=23<br>Mean age (SD)=35.26 (5.77)	Mean (SD)<br><br>Intervention: 56.27 (14.83)<br><br>Control: 62.73 (12.60)	None	None
Wayne et al., 2008<br>USA	RCT<br><br>Assess feasibility, and	Intervention: Japanese-Style Acupuncture, 16 acupuncture	Intervention:<br>n=9<br>Mean age (SD) = 17.8 (2.1)	Means (SD)<br><br>Intervention: 36.5 (20.2)<br>Control: 44.9 (16.5)	None	None

Author, year, country	Design / Purpose	Intervention or exposure potentially associated with QoL / QoL score and data point	Participants (n, age)	Level of quality of life at baseline	Factors significantly associated with improved quality of life	Factors significantly associated with a deterioration in quality of life
	collect preliminary data for a subsequent randomized, sham-controlled trial to evaluate Japanese-style acupuncture for reducing CPP and improving health related HRQOL in adolescents with endometriosis.	treatments; 2 per week for 8 consecutive weeks<br><br>Control: Sham acupuncture (designed to mimic active treatments, while being minimally active)<br><br>EHP-30<br>Baseline | 4 weeks | 8 weeks | 6 months following commencement of acupuncture treatments (8 weeks)	Control:<br>n=5<br>Mean age (SD) = 17.0 (2.1)			

Footnote: BMI = body mass index; CI = confidence interval; CPP = chronic pelvic pain; HRQoL = health-related quality of life; QoL = quality of life; MD = MD; NA = not answered; NRS = numeric rating scale; UK = United Kingdom; USA = United States of America; RCT = randomized controlled trial; SD = standard deviation; VAS = visual analogue scale. Bold is for significant associated factors.
